# MICRA: an automatic pipeline for fast characterization of microbial genomes from high-throughput sequencing data

**DOI:** 10.1186/s13059-017-1367-z

**Published:** 2017-12-19

**Authors:** Ségolène Caboche, Gaël Even, Alexandre Loywick, Christophe Audebert, David Hot

**Affiliations:** 1 University of Lille, CNRS, Inserm, CHU Lille, Institut Pasteur de Lille, U1019-UMR 8204-CIIL-Centre d’Infection et d’Immunité de Lille, F-59000 Lille, France; 2 Genes Diffusion, 3595, Route de Tournai, 59501 Douai, France; 3 PEGASE-Biosciences, Institut Pasteur de Lille, 1 Rue du Professeur Calmette, 59019 Lille, France

**Keywords:** Bioinformatics pipeline, Comparative genomics, Microbial genome characterization, High-throughput sequencing

## Abstract

The increase in available sequence data has advanced the field of microbiology; however, making sense of these data without bioinformatics skills is still problematic. We describe MICRA, an automatic pipeline, available as a web interface, for microbial identification and characterization through reads analysis. MICRA uses iterative mapping against reference genomes to identify genes and variations. Additional modules allow prediction of antibiotic susceptibility and resistance and comparing the results of several samples. MICRA is fast, producing few false-positive annotations and variant calls compared to current methods, making it a tool of great interest for fully exploiting sequencing data.

**Electronic supplementary material:**

The online version of this article (doi:10.1186/s13059-017-1367-z) contains supplementary material, which is available to authorized users.

## Background

High-throughput sequencing (HTS) technologies have emerged as a cost-effective and convenient approach for addressing many microbiological questions, drastically transforming this field. Having access to complete genomic information has revolutionized fundamental research in microbiology, allowing, for example, the development of novel antimicrobial compounds and vaccines [1]. HTS also enables new approaches for clinical applications, providing novel methods for genome-based diagnosis [2] and thus for treatment of infectious diseases [3] and outbreak follow-up [4]. The launch of second generation benchtop sequencers, cheaper platforms with performance that is adequate in terms of throughput for investigating microbial genomes, has contributed to this revolution in microbiology, and the coming third generation of sequencers should accelerate this. The Food and Drug Administration (FDA) is developing concepts for validation of HTS tests for infectious disease diagnostics and the detection of antimicrobial resistance and virulence markers [5], representing a milestone for HTS-based diagnostics. However, to be implemented in laboratories, standardization, optimization, and validation assays must be conducted for both the technical aspects and the bioinformatics analyses.

Despite the availability of a huge number of tools, analyzing HTS data can be challenging for fundamental and clinical research. The constant evolution of the technologies and algorithms result in a lack of standardized procedures for the analysis of HTS [6]. In addition, several tools have to be used successively for complete analysis, which is a difficult task without bioinformatics support. Only a few complete pipelines exist and most of them are only for pathogen identification. The term “identification” has to be distinguished from the term “characterization”. The aim of identification is to determine the strains, whereas the characterization of microbial genomes is an in-depth study to highlight genetic features, including gene transfer or infra-specific variation. A fast and automatic tool offering an easy-to-use interface and readable results for the characterization of microbial genomes could be of great interest for time-effective, full exploitation of HTS data.

Here we describe MICRA, for Microbial Identification and Characterization through Reads Analysis. The originality of MICRA lies in the way it exploits the increasing number of sequenced microbial genomes combined with efficient read mapping methods, rather than following the prevalent procedure of de novo assembly and sequence annotation. Big variations in sequencing depth—for example, if bacteria contain plasmids—could make the de novo assembly process difficult and result in a significant increase of the execution time. In MICRA, two mappers, a fast mapper and a robust mapper, are combined and a new adapted and fast variant caller specific for prokaryotes was developed, in order to be effective and to avoid any loss of data [7]. MICRA is freely available as a web interface developed to be user-friendly for both clinicians and biologists [8]. Parameters are optimized to offer the user an entirely automatic analysis, requiring only reads as input. For more specialized tasks, MICRA offers the possibility of customizable analyses by giving access to a lot of setting parameters. The outputs are directly readable and usable. MICRA was evaluated on several studies. All results show that MICRA is fast (around 10 minutes in most cases) and efficient and provides new insights for fundamental microbiology and for the study of circulating and emerging strains.

## Results

### The MICRA pipeline

**Fig. 1:**
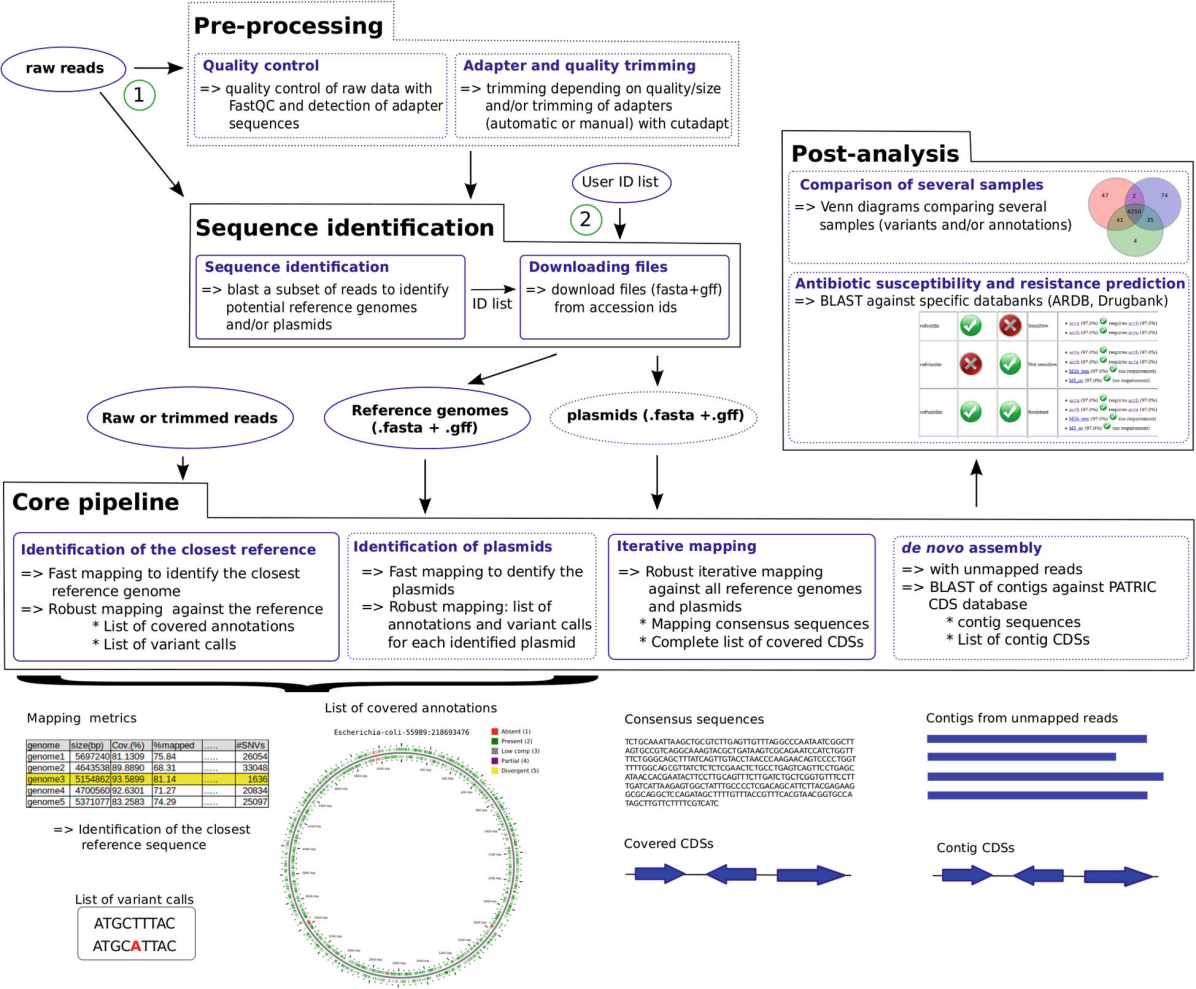
The MICRA pipeline. *Ovals* represent the input files. *Black boxes* represent the four main parts of the MICRA pipeline and *blue boxes* show the constitutive modules. *Dashed lines* are used for optional steps. *CDS* coding sequence

The structure of the MICRA pipeline, organized in four main parts, is schematically presented in Fig. 1.

The pre-processing part, which is optional, allows read quality checking and trimming. The first module uses FastQC [9] to check the quality of reads. Cutadapt [10] is used in the second module for adapter and quality trimming. Detection and trimming of adapters can be done automatically (sequence adapters are identified from FastQC results) or from sequences provided by the user. The reads are trimmed according to quality (Q20 by default) and size (20 nucleotides by default) thresholds; these values can be changed.

The sequence identification part aims to identify the reference sequences which will then be used in the core part of MICRA. The user can choose either to provide their own list of reference sequences (using NCBI identifiers; circle 2 in Fig. 1) or to let MICRA automatically select the potential reference sequences from reads (circle 1 in Fig. 1). To identify reference genomes and/or plasmids, a subset of the reads is BLASTed against a database containing selected complete genome and/or plasmid sequences (see “Methods”). The sequences showing the greatest numbers of hits are kept in an ID list. The sequence identification strategy was validated using several bacterial strains and sequencing technologies (Additional file 1: part 1). The second module automatically downloads FASTA and GFF files from NCBI or a local server for each entry in the ID list.

The core pipeline part of MICRA, consisting of four modules, takes as inputs the GFF and FASTA files obtained from the previous part and the FASTQ file(s) containing reads. In the first module, the whole set of reads is mapped against all the reference genome sequences with a fast mapper, SNAP [11]. Mapping metrics are returned for each reference sequence and the percentage of genome coverage value is used to identify the closest reference sequence. Then a robust mapper, SHRiMP2 [12] for Ion Torrent reads and Bowtie2 [13] for Illumina reads, is used with the whole set of reads against the closest reference genome, allowing the variant calls and a comparative list of annotations to be obtained (in CSV and FASTA format). For paired-end data, only the concordant pairs of reads are mapped in order to keep reads as pairs in the next steps. Home-made scripts were developed to compute the mapping metrics, the list of annotations, and to call variants from the SAM alignment file (see “Methods”). A SVG picture of the comparative annotations against the closest reference genome is also produced in this step using CGView [14]. Each feature from the annotation file is then classified into five classes by MICRA: 1) absent (less than 10% covered), 2) present (more than 80% covered), 3) low complexity sequence (detected with DustMasker [15]), 4) partially present (coverage between 40 and 80% distributed among up to two blocks of conservation), 5) divergent (otherwise). When the user asks for plasmid processing, the same steps are performed leading to the identification and characterization of plasmids. In the third module, iterative mapping with the robust mapper is performed: the reads which are not mapped against the closest reference genome and plasmids are then iteratively mapped against the other reference genomes. This step produces consensus sequences built from the mapping (i.e., for consecutive covered positions in the reference sequence the consensus sequence is deduced from the reads) and the complete list of covered annotations. The reads that are still unmapped at the end of the iterative mapping steps are then used in the de novo assembly module. MIRA [16] is used for de novo assembly of Ion Torrent reads and SPAdes [17] for Illumina reads. Resulting contigs are then annotated by BLASTing them against the PATRIC CDS database [18]. Sequences and annotations produced by the core part of MICRA can then be used in the post-analysis part.

The post-analysis part contains two independent modules. The comparison module allows the user to compare the annotations and/or the variant calls from several samples. A Venn diagram is produced to easily compare the different samples; the genes or variants from the different parts of the Venn diagram are also available. The second module aims to identify potential antibiotic susceptibility and resistance. Susceptibility and resistance genes for a given list of antibiotic drugs can be detected from the sequences produced by the core pipeline. The susceptibility genes are identified by BLASTing the sequences against a local version of DrugBank [19] and the resistance genes are identified by BLASTing the sequences against a modified version of the Antibiotic Resistance DataBase [20] (see “Methods”). A very easy to interpret table (in HTML format) summarizing the predicted susceptibility and resistance for each drug is returned, as well as the detailed BLAST results.

MICRA is publicly available at http://www.pegase-biosciences.com/MICRA. The web interface was developed to be intuitive and easy to use: only the FASTQ file containing reads and the indication of the sequencing technology used are required. However, the MICRA interface also offers the user many customizable parameters to fit different requirements and all the cutoffs used in MICRA can be adjusted. Results from MICRA are returned as a zip directory containing a user-friendly summary HTML file allowing easy navigation of the results. A detailed user guide describing the parameters and the generated result files is available on the website [21].

### Validation studies

In order to validate the MICRA pipeline, we first used the well-studied genome of *Escherichia coli str. K12 substr. DH10B* under fully controlled conditions with real and simulated reads. Promising results were obtained and confirmed in two real context studies: a fundamental research case study with a strain of *Bordetella pertussis* (which has a genome with a high number of repeats) and a clinical case study with the data from a German outbreak caused by an unusual strain of *Escherichia coli*.

### Formal validation of MICRA using *Escherichia coli str. K12 substr. DH10B* data

In order to test MICRA under fully controlled conditions, simulated reads were generated from a genome artificially mutated by creation of base variations, gene insertions, and deletions. The genome of *E. coli str. K12 substr. DH10B* was artificially mutated: 23,290 point mutations were randomly introduced along the genome sequence, two shiga toxin genes (Shiga toxin 2 subunit A and B) from *E. coli O157:H7 str. Sakai* were inserted, and finally the *mdtL* gene, implicated in chloramphenicol resistance, was deleted (see “Methods”). From this artificially mutated genome sequence, one million artificial reads with a mean length of 200 bp and mimicking Ion Torrent read characteristics were simulated using CuReSim [7] and paired-end (PE) Illumina reads of 100-bp length and DNA fragments with a mean size of 200 bp (standard deviation of 10) were simulated with ART [22] to obtain a theoretical sequencing depth around 40×. To mimic a plasmid transfer event, reads simulated from the pSFO157 plasmid to reach a theoretical mean sequencing-depth of 80× were added.

MICRA was used with the FASTQ file(s) containing the simulated reads. The *E. coli str. K12 substr. DH10B* genome (NCBI: CP000948.1) was identified as the closest reference genome, covered at 99.94% (99.971% for PE reads) with a mean depth of 42× (39.99× for PE reads). Observing the comparative annotations of K12-DH10B, only one coding sequence (CDS) was tagged as absent, corresponding to the artificially deleted gene *mdtL*. A total of 20,571 variant calls (20,834 for PE reads) were predicted and 20,558 (20,833 for PE reads) of these were correctly predicted, corresponding to a recall of 0.882 (0.894 for PE reads) and a precision of 0.999 (0.999 for PE reads). In order to validate our variant caller, these results were compared with the results produced by freebayes [23], an independent variant caller (see “Methods”): 20,153 (20,971 for PE reads) variants were predicted in around 5 minutes, leading to a recall of 0.864 (0.900 for PE reads) and a precision of 0.999 (0.999 for PE reads). These results prove that the in-house variant caller is efficient and fast.

The plasmid pSFO157 (NCBI: AF401292.1) was identified by MICRA with a coverage of 97.18% (98.20% for PE reads) and a mean depth of 78× (78× for PE reads), close to the theoretical expected sequencing depth. The comparative genomics results from the mapping against the pSFO157 plasmid showed that two CDSs (w0034 and w0076) were not covered. These two CDSs code for a duplicated IS30 transposase gene. This gene sequence is also present in the K12-DH10B genome, resulting in the mapping of the corresponding reads onto the genomic IS30 transposase sequence during the first step of the pipeline analysis and explaining the non-covered region in the plasmid sequence. The consensus sequence built from the mapping step produced six sequences (three with PE reads) from K12-DH10B and eight sequences (three with PE reads) from the pSFO157 plasmid. At the end of this step, 415 reads (1334 pairs) were still unmapped and used for de novo assembly, producing only one contig (one contig for PE reads) larger than 500 bp. For both single and paired-end read simulations, automatic annotation of the de novo generated contig identified CDSs, among which were the Shiga-like toxin II subunit A precursor and Shiga-like toxin II subunit B precursor. The de novo assembly step allows MICRA to detect the artificial horizontal transfer of the two shiga-toxin genes. The antibiotic susceptibility and resistance module highlighted two differences in comparison with the prediction from the K12-DH10B genome sequence: the artificial strain is predicted to be sensitive to chloramphenicol due to the deletion of the *mdtL* gene and to be tetracycline-resistant due to a sequence encoded in the artificially introduced pSFO157 plasmid.

In order to avoid simulation biases and confirm the previous results, the same experiment was carried out after changing the initial conditions: one million real Ion Torrent reads (to reach a mean depth of 40×) were used as input and the artificially mutated genome sequence was integrated among the reference genomes instead of the K12-DH10B genome. MICRA identified the K12-DH10B mutated genome as the closest reference with a coverage of 99.92% and a mean depth equal to 38×. The only CDSs not covered were shiga toxin subunit A and B, which is consistent with the fact that shiga toxin genes are not naturally present in the K12-DH10B genome. Out of the 22,741 total predictions, 22,733 mutations were correctly predicted, corresponding to a precision of 0.999 and a recall of 0.976 (0.999 and 0.938 respectively with freebayes). Better recall values were obtained with the real reads rather than with the simulated ones, certainly due to higher error rates in the simulated data [7]. The *mdtL* gene, which was deleted in the mutated genome, was identified during the iterative mapping against the MG1655 genome (NCBI:NC_000913.3). The de novo assembly with the 11,944 remaining unmapped reads resulted in eight contigs greater than 500 bp corresponding to human sequences. The antibiotic susceptibility and resistance module used with these data showed a profile identical to the expected wild strain profile with chloramphenicol resistance mediated by the *mdtL* gene and tetracycline susceptibility.

MICRA was then assessed under real-life conditions and results were compared to external tools. A set of 2,290,055 reads with a mean length of 200 bp and a mean quality of Q28 from a 316 Ion Torrent run was used [24]. First, MICRA was used with the entire set of reads and only the K12-DH10B genome (NCBI:CP000948.1) as reference sequence. This illustrates how variation between a mutant and its related wild-type strain can be identified. The reference genome was covered at 100% with a mean depth of 88×, producing a complete consensus sequence from the mapping step. Two variants were called by MICRA: one SNV of a T to a C at position 1,103,559 with a frequency of 90.14% and a depth of 96 reads at this position in an IS1 transposase (locus: ECDH10B_1059), implying an amino acid change from a tyrosine to a histidine; and one insertion of a T at position 4,272,971 with a frequency of 95.33% and a depth of 107 reads at this position. Sanger sequencing of these two regions was performed (see “Methods”). The insertion of a T at position 4,272,971 was confirmed, but the SNV was not. When the 200-bp sequence around the false positive SNV was searched for in the genome, eight repeated regions were identified. Seven of the eight repeats contained a C whereas one repeat contained a T. The mapper was not able to discriminate the reads in these regions and mapped them on the eight possible positions. In position 1,103,559, the real base is a T but the pipeline flagged a mutation to a C due to the multi-position mapped reads. De novo assembly of unmapped reads led to 26 contigs greater than 500 bp. Those sequences matched mainly with human repeated sequences.

Secondly, to evaluate the pipeline behavior without the K12-DH10B reference sequence, the MICRA sequence identification module was first run to select six genomes and five plasmids in order to obtain a final list of reference sequences containing five genomes (excluding the K12-DH10 genome) and five plasmid sequences. The closest reference genome identified was *E. coli str. K12 substr. MG1655* (NCBI: NC_000913.3) with a coverage of 97.41%, a mean depth of 88×, and around 98% of reads mapped. No plasmids were identified but 4038 CDSs were predicted from the *K12 substr. MG1655* genome, and 14 from the other reference genomes. De novo assembly produced 21 contigs greater than 500 bp. Five of the longest contigs corresponded to *K12-DH10B* sequences and are specific to this strain. The other contigs correspond to human sequences. In order to evaluate the pipeline performance, the MICRA results were compared with de novo-based approaches. MIRA [16] and IonGAP [25] were used with the same set of reads and QUAST [26], a quality assessment tool for genome assembly, was used to compare the results which are shown in Table 1.

**Table 1:** Comparison of results obtained with MICRA, MIRA and IonGAP

	MICRA with DH10B	MICRA without DH10B	MIRA	IonGAP
Time	9 minutes	11 minutes	3 h	>3 h
Number of contigs > 500 bp	1	41	267	197
N50	4686138	2836109	88618	121061
Genome fraction (%)	100	97.195	96.314	96.752
Number of Ns	2	91	89	4
Number of mismatches	1	73	147	116
Number of short indels	1	17	63	64
Number of long indels	0	5	5	0
Number of genes	4127 + 0 part	4019 + 7 part	3919 + 106 part	3927 + 95 part
Number of misassemblies	0	19	5	9

The first interesting difference is the runtime: more than 3 h were required to produce an assembly with de novo-based approaches whereas MICRA required around 10 minutes. With the reference genome, MICRA was able to reconstruct 100% of the genome. When the K12-DH10B genome was discarded, MICRA was still able to cover 97% of the genome sequence with 41 contigs covering 4019 complete and seven partial genes on the 4127 genes annotated in the K12-DH10B genome (NCBI:CP000948.1), with a reduced number of false positive mutations (73 mismatches and 22 indels). De novo assembly-based approaches produced at least four times more contigs than the MICRA approach (267 with MIRA and 197 with IonGAP), covering around 96.5% of the reference sequence and a lower number of covered genes. The number of errors is greater than the number obtained with MICRA, especially for indels. IonGAP used MIRA for the assembly steps, keeping only large contigs (>500 bp), so the results are close to the results obtained with MIRA only. MICRA sequences contained more Ns than the assembly-based methods due to the low coverage positions which are encoded by N in our method. The number of miss-assemblies was greater for MICRA and were mainly relocations. Relocation is a miss-assembly where the left flanking sequence aligns over 1 kbp away from the right flanking sequence on the reference, or they overlap by more than 1 kbp, and both flanking sequences align on the same chromosome. The MICRA pipeline used a reference genome to obtain consensus sequences and the order of the gene on the reference genome can differ from the sequenced genome, producing a relocation event in this evaluation. In addition to the assembly step, IonGAP uses a comparative genomics module and a module for bacterial classification and annotation, producing some results directly comparable with the MICRA results. IonGAP contains a variant caller using raw reads and requiring a reference genome, here K12-DH10B, making this step comparable to the MICRA results with the reference genome. Two mutations were detected with MICRA, one true positive and one false positive. With IonGAP, 2684 mutations were predicted, of which 18 with genotype confidence equal to or greater than 10 could be considered as significant. Among the 18 mutations, 17 were tested by Sanger sequencing and proved to be false positives, and the true positive mutation, the insertion of a T at position 4,272,971, was not identified. No plasmid sequences were present in the sequencing sample. MICRA predicted no plasmids but IonGAP returned 260 BLAST hits for plasmid identification.

### A fundamental research case study: the Pillemer strain of *Bordetella pertussis*

*Bordetella pertussis* is a pathogen restricted to humans. It is the causative agent of whooping cough. In 1954, the first acellular vaccine against pertussis was introduced by Louis Pillemer using the strain which was named after him, Pillemer (or P134). Ten years later the use of this strain as a vaccine was abandoned because of toxicity problems and it was replaced by whole cell vaccines. The genomic characteristics of the Pillemer strain have only been superficially investigated, mainly using PFGE (Pulsed-field gel electrophoresis) profiles for comparison to other circulating or vaccinal strains in neighbor-joining classification studies [27]. We sequenced a lab-adapted Pillemer strain (named P134S for streptomycin resistant) using a PGM Ion Torrent sequencer [28]. The genomes of *B. pertussis* strains contain a high number of inserted repeated sequences (more than 260 for the Tohama I strain) and we therefore used the MICRA options for analysis of high-repeat content genomes (i.e., the parameters of the mapper are adapted to fit the high-repeat content). As expected, *Bordetella pertussis* Tohama I strain (NCBI:NC_002929.2) appears to be the closest reference genome, with a coverage of nearly 94% and a mean depth of sequencing close to 17× (Additional file 1: part 2-1). The variant calls between P134S and Tohama I (Additional file 1: part 2-2) showed the low number of only 351 variations (304 SNVs and 47 indels). To evaluate the reliability of the MICRA variant caller we experimentally confirmed five out of the 351 variations: three coding SNVs, one non-coding SNV, and one insertion were tested by Sanger sequencing (Additional file 1: part 2-3). These five variations were confirmed at the exact positions detected by MICRA and the predicted inserted bases in *fimC* were confirmed to be a TG dinucleotide as predicted.

**Fig. 2:**
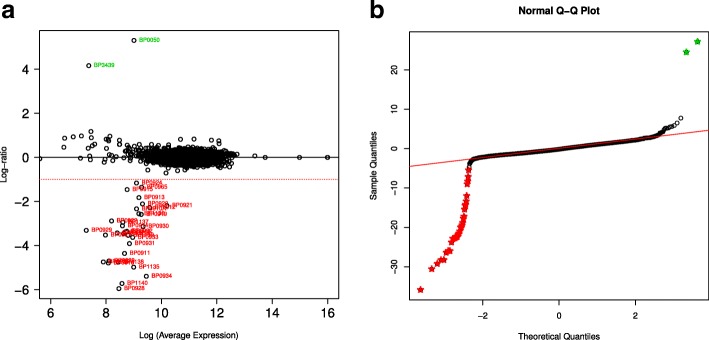
CGH array results comparing the P134S strain gene content to BPZE1 gene content. **a** Obtained log ratios from the CGH array experiment are represented as a MA plot. Genes within the clusters predicted by MICRA to be absent in P134S are indicated in *red*. Genes known to be absent in BPZE1 are indicated in *green. Dashed red line* represent log ratio = −1. **b** QQ plot of t-statistic for P134S strain gene content compared to BPZE1 gene content as determined by CGH array. Quantiles of t-statistics distribution for all ORFs were plotted against a normal standard distribution. Genes highlighted in panel **a** are marked with *red* and *green stars* and appear not normally distributed, indicating a highly divergent log ratio

MICRA also detected two clusters of Tohama I genes absent in the P134S genome (from BP0911 to BP0934 and BP1136 to BP1141). A CGH array experiment using a microarray of the whole coding genes according to annotation file NC_002929 was performed as previously described [29]. This CGH assay compared the P134S genome, as query template, to a derivative of Tohama I strain called BPZE1 [30] as reference. BPZE1 was used instead of the Tohama I genome because two genes (*dnt* and *ampG*) were artificially removed and can therefore be considered as an internal control of the CGH array reliability. The log ratios of hybridization intensities between P134S and BPZE1 genome-derived targets were calculated. As expected, the log ratios of known absent genes in BPZE1 (*dnt* and *ampG*) are significantly very high, indicating their presence in P134S and absence in BPZE1. In contrast, the log ratios of intensities for the reporters of clusters BP0911 to BP0934 and BP1136 to BP1141 show significantly low values (logFC < −1), indicating absence of these genomic regions in the PP134S genome (Fig. 2a). This analysis was validated by comparing the calculated t-values to the normal distribution using a QQ plot representation. Divergent genes show clear discrimination on the QQ plot, representing a not normally distributed group on the left-hand side of the graph for the genes absent in the P134S strain and on the right-hand side for the genes absent in BPZE1 (Fig. 2b).

Finally, four additional regions were detected by MICRA in the P134S genome compared to Tohama I. These regions were detected to be highly similar to four clusters of genes present in the *Bordetella bronchiseptica* RB50 genome. The presence of these regions in P134S was validated by PCR (Additional file 1: part 2-4).

### A clinical case study: the 2011 German outbreak caused by *E. coli* O104:H4

In May of 2011, an enteroaggregative *E. coli* O104:H4 strain disseminated through contaminated bean sprouts caused a large outbreak in Germany [31]. This strain caused hemolytic, uremic, and enterohemorrhagic diarrhea syndrome and a total of 4075 cases and 50 deaths were reported. Initially, the isolate from a 16-year-old girl (TY2482) was sequenced using the Ion Torrent PGM and five runs on 314 chips were produced. Illumina sequencing was then performed to obtain the finished complete sequence of a TY2482 strain that contained one chromosome and three plasmids (pG, pESBL, and pAA) [32]. Sequencing data from these circulating strains represented good material to evaluate the pipeline performance in the context of a real infectious case. In all experiments, we only used the five first runs generated with the Ion Torrent technology [33] in order to simulate the situation at the early stage of the outbreak period. The poor quality of the sequencing data is due to the early age of the technology at that time.

**Fig. 3:**
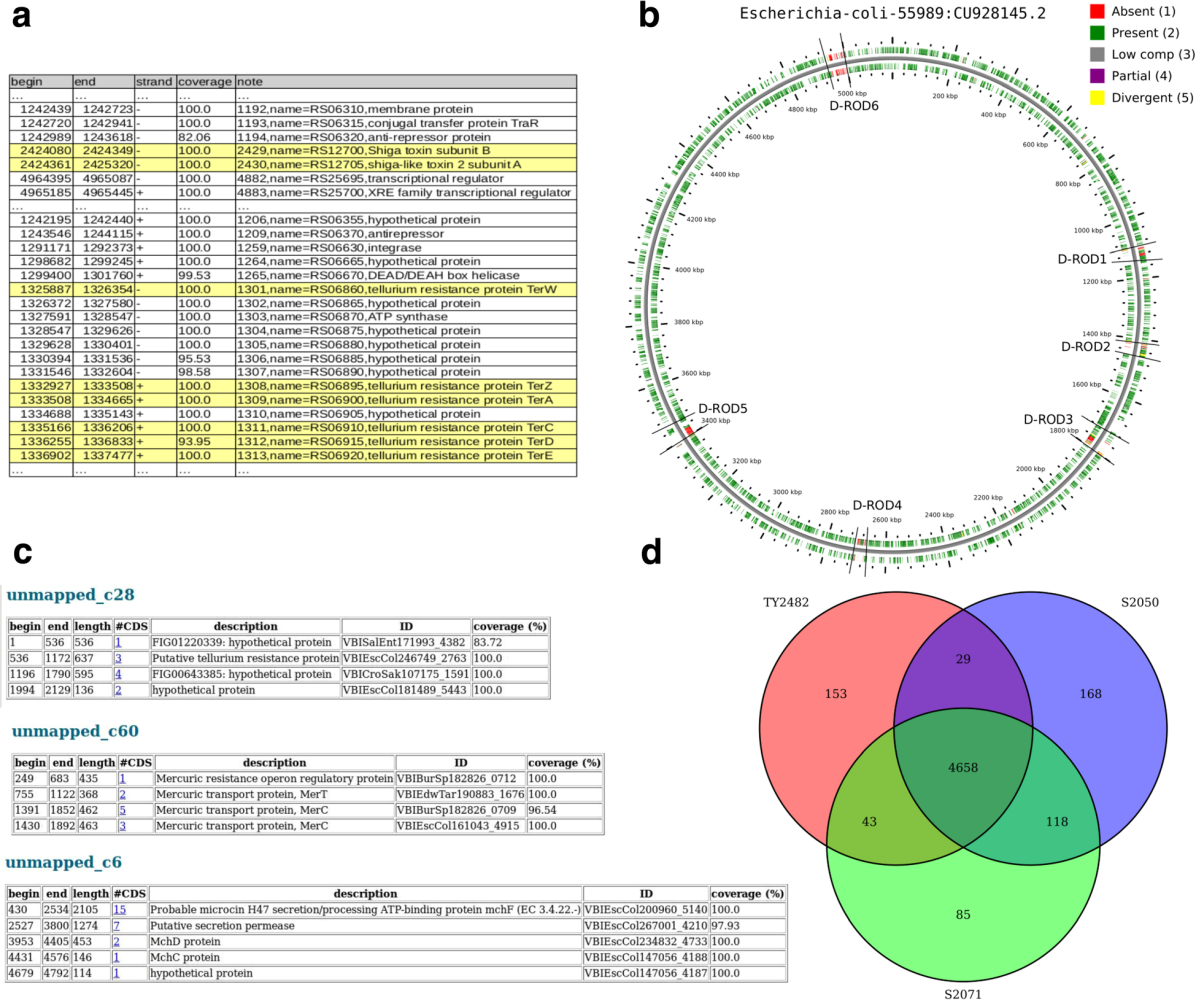
Examples of MICRA results highlighting the biological features. **a** Extracted lines of the CSV annotation file from mapping. The *yellow highlighted lines* show the shiga toxin 2 genes and several tellurite resistance genes characteristic of the strain. **b** The comparative genome picture produced with CGView against the *E. coli 55989* strain. The additional ROD elements correspond to the deleted regions identified in Rhode et al. [32]  **c** Extracted results of MICRA de novo contig annotation showing components of the microcin gene cluster, the tellurite resistance gene cluster, and the mercury resistance gene cluster. **d** Venn diagram showing the comparison of CDSs obtained with MICRA between the strains EL2009-2050, EL2009-2071, and TY2482

MICRA was first used with the TY2482 chromosome and plasmids to evaluate the performance of the analysis, independent of the choice of the reference sequences given as input (Additional file 1: part 3-1). MICRA was then run in an automatic fashion. In order to get as close as possible to the original outbreak conditions, the sequences released after 2011 were discarded from the MICRA database, leading to the automatic selection of five genomes and ten plasmids. The closest reference genome identified was *E. coli* strain 55989 (NCBI:CU928145.2), covered at more than 95% with a mean depth of 10×. Two plasmids were identified: a plasmid of *E. coli O7K1 str. CE10* (NCBI:CP003038.1) similar to the pG plasmid with a coverage of 100% and a mean depth of 413×, and the plasmid pEC Bactec of *E. coli* (NCBI:GU371927.1) similar to the pESBL plasmid and covered at 96% with a mean depth of 11×. No plasmid similar to pAA was identified due to the similar region shared between this plasmid and the chromosome sequence. During the analysis, the strain was rapidly characterized and we checked that the main features could be easily retrieved in the MICRA results. Figure 3 shows some MICRA results highlighting the features described in [32]*.*

The MICRA pipeline, for the main part, is not based on de novo assembly, which is a process known to be time-consuming. We compared the MICRA results with those obtained with the existing de novo-based approaches. QUAST [26] was used to compare the sequences obtained with MICRA, IonGAP, and MIRA (Additional file 1: part 3-2). The number of contigs produced by MICRA is lower, the N50 is greater, and the genome fraction is similar between the two approaches. MICRA induced more mismatches than the de novo approaches but much fewer indels, which is the main error type induced by Ion torrent technology but are well taken into account by MICRA.

Another point to be considered with de novo-based approaches is that genes have a high probability to be fragmented into several contigs, making the sequence annotation difficult. To evaluate this bias, the performance of MICRA was compared to results obtained from a de novo assembly with three different prokaryotic annotation tools: BG7 [34], developed for analysis of this infectious episode, RAST [35], and PROKKA [36]. The results from IonGAP, based on a de novo assembly with MIRA and PROKKA for the annotation step, were also included. Table 2 shows the precision, recall and F-measure values obtained with the five approaches. MICRA predicted fewer CDSs than any of the other tools but the precision value was significantly better with MICRA (0.95), meaning that the CDSs are well predicted (few false positives) and its recall value (0.87) was close to the other tools. These results show that our method based on mapping and comparative genomics allows for the correct detection of a large part of the gene content of the sequenced organism, in a much shorter time.

**Table 2:** Precision, recall, and F-measure values for five sequence annotation approaches

	Number of CDSs	True positives	False negatives	False positives	Precision	Recall	F-measure
MICRA	4913	4658	695	255	0.95	0.87	0.91
BG7	6190	4846	507	1344	0.78	0.91	0.84
RAST	7958	4778	575	3180	0.6	0.89	0.72
PROKKA	7482	4840	513	2642	0.65	0.9	0.75
IonGAP	6514	4411	942	2103	0.68	0.82	0.74

The Robert Koch Institute provided the antibiogram for this strain that we compared to the antibiotic susceptibility profile predicted by MICRA and with the results obtained with two other methods: results from Kuznetsov et al. [37], consisting of BLASTing contigs against ARDB and DrugBank, and ResFinder [38]. IonGAP results were not included in the comparison because they are raw hit BLAST and a link between an alignment and the corresponding drug resistance cannot be easily made. Table 3 shows the results of the predictions. MICRA correctly predicted 20 of the 22 in silico predictable antibiotics. The incorrect prediction for chloramphenicol is due to the detection of the *mdtM* and *mdtL* genes, which encode multidrug-resistance proteins associated with chloramphenicol resistance but are not included in ResFinder. In comparison, the other tools did not make as many predictions, 15 for Kuznetsov et al. and 17 for ResFinder, with two incorrect predictions for both of them. Results showed that MICRA is therefore able to predict a susceptibility profile close to the real antibiogram.

**Table 3:** Comparison between experimentally measured and computationally predicted antibiotics susceptibility profiles

Antibiotic	Class	Exp.	MICRA	Kuznetsov et al.	ResFinder
Ampicillin	Beta-lactam, penicillins	R	R*	ND	R**
Amoxicillin	Beta-lactam, penicillins	R	R*	ND	R**
Piperacillin	Beta-lactam, penicillins	R	R*	**S**	R**
Cefuroxim	Beta-lactam, cephalosporins	R	R*	ND	R**
Cefoxitin	Beta-lactam, cephalosporins	R	R	R	R**
Cefotaxim	Beta-lactam, cephalosporins	R	R*	ND	R**
Ceftazidim	Beta-lactam, cephalosporins	R	R	R	R**
Cefpodoxime	Beta-lactam, cephalosporins	R	R	R	R**
Imipenem	Beta-lactam, carbapenems	S	S	S	R**
Meropenem	Beta-lactam, carbapenems	S	S	S	R**
Amikacin	Aminoglycoside	S	S	S	ND
Gentamicin	Aminoglycoside	S	S	S	ND
Kanamycin	Aminoglycoside	S	S	S	ND
Tobramycin	Aminoglycoside	S	S	S	ND
Streptomycin	Aminoglycoside	R	R	R	R
Ciprofloxacin	Fluoroquinolone	S	S	ND	S
Norfloxacin	Fluoroquinolone	S	R*	ND	S
Tetracyclin	Polyketide	R	R	R	R
Nitrofurantoin	Furans	S	S	S	ND
Trimethoprim/sulfamethoxazole	Aminopyrimidine	R	R	R	R
Chloramphenicol	Phenicol	S	**R**	ND	S
Fosfomycin	Phosphonic acids	S	S	**R**	S

Note that ResFinder mainly makes predictions for antibiotic classes rather than individual drugs. *R* resistant, *S* sensitive, R* not sensitive considered as a particular resistance, R** prediction for beta-lactam class, not individual drugs, *ND* not determined. Incorrect predictions appear in bold text

Two *E. coli O104:H4* isolates close to TY2482 were sequenced and studied [39]: 2009EL-2050 and 2009EL-2071. We used MICRA with a subset of the single Illumina reads and the same reference genomes as the sequences used for the TY2482 isolate (Additional file 1: part 3-3). MICRA identified a new plasmid close to the *E. coli LF82* plasmid for the 2009EL-2050 strain, which is specific to this strain [39]. An interesting result was a difference between the two predicted resistance profiles: 2009EL-2050 was predicted to be resistant to the tetracycline whereas the 2009EL-2071 strain was predicted to be sensitive, which was observed by experimentally measured antibiotics resistance profiles [39]. The MICRA comparison module was then used to rapidly compare the TY2482, 2009-2050, and 2009-2071 strains. A Venn diagram (Fig. 3d) showed that the three strains share a large number of CDSs and that the two 2009EL strains share more CDSs. Among the CDSs shared between TY2482 and 2009EL-2050, the *tetA* gene implicated in the tetracycline resistance was retrieved by MICRA.

### Additional application examples

In order to confirm the performance of MICRA, we performed two additional complete case studies with Ion torrent and Illumina paired-end data from *Staphylococcus aureus* and *Clostridium autoethanogenum* (Additional file 1: part 4). The results show that MICRA produced redundant sequences with paired-end data, especially with selected sequences genetically distant from the studied sequence, but also that it is still able to correctly identify more true CDSs than the other approaches. We also showed that MICRA is able to deal with varying sequencing depth and can be of great interest in cases where the assembly process is difficult (Additional file 1: part 4-1 on *S. aureus* Ion torrent data). Finally, the results greatly illustrate the potential and power of using MICRA in re-sequencing projects or mutant study projects (Additional file 1: part 4-2 on *C. autoethanogenum* data).

## Discussion

The accessibility of HTS technologies has revolutionized the field of microbiology. However, some challenges have to be addressed before HTS becomes a routinely used tool, especially because of data analysis, which is still lacking standard and easy-to-use protocols driven by robust pipelines [6]. In this view, we developed a pipeline for fast and efficient automatic characterization of microbial genomes from HTS data.

MICRA is able to deal with data produced by the two main benchtop sequencers (Ion Torrent PGM and Illumina MiSeq). It easily and rapidly highlights interesting biological and clinical features, predicts antibiotic susceptibility and resistance profiles, as well as compares several strains in a matter of minutes. We tested MICRA with several validation cases and, in all of them, MICRA determined gene content with result quality equal to or better than other existing solutions. The power of HTS analysis with MICRA is illustrated by the immediate determination of variation points compared to a reference strain, allowing the user to immediately focus on important variations which could explain phenotype differences. This rapidity and efficiency is of particular interest for the characterization of a large number of strains, for instance in a microbial GWAS context [40].

In the near future, we hope that MICRA will become enriched with additional modules thanks to user feed-back. MICRA was developed and optimized for bacterial genomes but can also be adapted to other organisms such as viral or small eukaryotic genomes. Due to its mapping iterative approach, MICRA should deal with sequenced genomes obtained from culture-independent methods.

To our knowledge, MICRA is the only automatic HTS pipeline based on mappers and comparative genomics, offering an easy-to-use web interface and readable results. MICRA is fast enough to be used in real-time outbreak contexts and entirely fits with actual needs for wider HTS use in microbiological and clinical research.

## Conclusions

Fast and cost-effective access to HTS data has prompted the development of efficient and reliable solutions for bioinformatics analyses. Here we present MICRA, an automatic pipeline for microbial genome characterization based on iterative read mapping onto annotated reference genomes available in databases. MICRA allows the rapid annotation of genomes and plasmids and the efficient determination of variations relative to close reference genomes thanks to the development of a new variant caller specific for prokaryotes. Additional modules allow for comparison of results and automatic determination of antibiotic resistance profiles. MICRA is freely available [41, 42] and easy-to-use as a web interface [8] requiring only reads as input. Assessment of MICRA in formal, clinical, and research contexts shows that it is much more rapid (10 minutes in most cases) and accurate than the usual de novo-based assembly methods.

## Methods

### Databases used in MICRA

The identification module of MICRA uses homemade databases containing some of the complete sequences extracted from the NCBI and selected for their completeness, for the quality of their annotations, and to avoid similar sequences. We extracted 1533 complete genomes from the ftp summary file for bacterial genomes [43].

Similarly, an initial list of 4437complete sequences for plasmids was obtained from [44]. However, some plasmid sequences were very similar. We used PSI-CD-HIT [45] (options -c 0.9 -G 1 -g 1 -prog blastn -circle 1) to cluster sequences showing more than 90% identity and we kept only one representative sequence of each cluster, leading to a final database containing 3807 complete plasmid sequences.

The PATRIC CDS database is used during the annotation of the contigs produced by de novo assembly.

The antibiotic susceptibility and resistance module used two databases: the susceptibility is predicted from a local version of DrugBank (http://www.drugbank.ca) and the antibiotic resistance determination is based on a modified version of ARDB (https://ardb.cbcb.umd.edu/). Some links between resistance genes and drugs were added and some others were deleted by comparing the genes and associated drugs with The Comprehensive Antibiotic Resistance Database (CARD) [46]. The beta-lactams were re-encoded according to CARD data and the fosfomycin link was deleted in the deoxycholate association from ARDB.

### MICRA scripts and external software

MICRA contains in-house scripts developed in Java for running, linking, and parsing the results of the steps executed with external software [41, 42]. In addition to these constitutive scripts, a variant caller was developed to be accurate, fast, and adapted to prokaryotic genomes. The reference sequence is encoded by an array and for each position the mapping data are stored: the corresponding base in the reference genome, the number of reads mapped at this position (a multi-mapped read is counted as 1/n with n the number of mapping positions for this read), and the list of variants with their frequency. The consecutive variant positions are not considered independent: when a deletion of three consecutive bases occurs the mutation is not counted on the three corresponding bases but as a deletion of size 3 in the first position, which decreases the number of false positive variant calls. In addition to call variants, data encoded in the array-based data structure also allow the genome coverage, the mean depth, and the coverage in percentage of annotations to be computed.

MICRA is using several other technologies and external software in its different parts: FastQC [9] and cutadapt [10] in the pre-processing part, BLAST 2.2.28 [47] in the sequence identification part, core pipeline and antibiotic parts, BioPerl in the sequence identification part to download files from NCBI, readSeq in the core pipeline to format FASTQ file, the bp_genbank2gff3.pl script [48] to convert GenBank format to GFF format in the sequence identification part, DustMasker [15] to identify low complexity regions in the core pipeline, SHRiMP 2.2.3 [12] for the robust mapping of the Ion Torrent reads, SNAP 0.15 [11] for the fast mapping of the Illumina and Ion Torrent reads, bowtie 2-2.2.9 [13] for the robust mapping of Illumina reads, MIRA 4.0.2 [16] for de novo assembly of Ion Torrent reads, SPAdes-3.9.0 [17] for de novo assembly of Illumina reads, CGView [14] for comparative genomics visualization. The comparison module is based on R with the use of the VennDiagram package [49] to produce graphics.

### The MICRA web interfaces and the computational resources

The MICRA Web service is deployed on an Ubuntu server (version 14.04) with 256 GB of RAM and 2 × 12-core processors (2.6 GHz). An Apache HTTP server with PHP and javascript technologies are used for the MICRA website. MICRA jobs are run in docker containers to eliminate conflicts and enhance security.

### Simulated data from the K12-DH10B genome

The genome of *E. coli str. K12 substr. DH10B* (NCBI:CP000948) was artificially mutated: 21,052 SNVs and 2238 deletion/insertion polymorphisms (DIPs) were randomly introduced using a script developed specifically to record position and nature of mutations. Two shigatoxin genes (Shiga toxin 2 subunit A and B) from *E. coli O157:H7 str. Sakai* (NCBI:NC_002695) were inserted between positions 1,557,859 and 1,557,915 and finally the *mdtL* gene implied in chloramphenicol resistance (3,987,222–3,988,397) was deleted. From this artificially mutated genome, 1,000,000 reads were simulated with CuReSim 1.2 to obtain a theoretical sequencing-depth around 40× with the default parameters (read size of 200 bp with a standard deviation of 20, 1% of deletions 0.5% of insertions, and 0.5% of substitutions). To mimic the transfer of a plasmid, 50,000 reads from the pSFO157 plasmid (NCBI:AF401292), corresponding to a theoretical sequencing-depth of 80×, were also simulated with CuReSim (default parameters) and added to the FASTQ file which was finally shuffled. Paired-end Illumina reads were simulated with ART from artificially mutated genome with parameters -l 100 (read size) -m 200 (mean fragment size) -s 10 (standard deviation for fragment size) and -f 40 (sequencing-depth of 40×) and from pSFO157 plasmid with parameters -p -l 100 -f 80 -m 200 -s 10. Resulting reads were then merged in two FASTQ files which were finally synchronously shuffled.

### Evaluation of the MICRA variant caller

To evaluate the MICRA variant caller, we compared our results to the variant calls with FreeBayes, a variant caller able to call variants from prokaryotic data. Freebayes was used in version 9.9.2-27 (commit id:5d5b8ac) with the default settings except the -p parameter set to 1 for haploid genome. FreeBayes produces a file in Variant Call Format (VCF) that contains all variations. The VCF file was filtered to keep only variations with a depth of at least 5 reads and a frequency of at least 90% to be comparable to the MICRA results.

The performance of the variant callers was evaluated by computing precision and recall values as follows: precision = CM/(CM + IM) and recall = CM/(CM + IM + NM), where *CM* is correctly identified mutations, i.e., same type and same or equivalent position as the artificially introduced mutation, *IM* is incorrectly identified mutation, and *NM* is not-found mutation.

### Sanger validation of the K12-DH10B variant calls

MICRA predicted SNVs for *E. coli str. K12 substr. DH10B* were targeted by PCR. Thus, two primer pairs were designed: one targeting the putative T/C transition with forward primer gtcatcgtctgcgcggaaatg and reverse primer gatctcaagcgtacgtattgtcggt, amplifying a locus of 433 bp (starting position 1103286 to ending position 1103718), the other targeting the putative T-insert with forward primer cccactggttgcggtcaaact and reverse primer tggtgccgactaccggaatcg, amplifying a locus of 497 bp (starting position 4272686 to ending position 4273182). Each PCR mix has been compounded with 300 nM of each primer, 2.5 U of HotStart Taq Plus (Qiagen, Venlo), 2 mM of MgCl_2_ and 100 ng of *E. coli* DH10B DNA extract. PCR occurred following thermocycling: 5 min at 95 °C as activation step and 36 cycles composed of a denaturating step of 15 s; at 95 °C, annealing of 15 s; at 58 °C, an extension of 1 min at 72 °C. A final extension step occurred during 4 min at 72 °C. PCR products were purified with a QIAquick PCR Purification Kit (Qiagen, Venlo) and then Sanger sequenced. In the same way, PCR/Sanger sequencing were carried out for each suspected false positive SNV found with IonGap with following primers:

### Sequencing data

The K12-DH10B reads were obtained from Life technology from an Ion Torrent run with a 316 chip. The corresponding FASTQ files are available at [24].

The Pillemer P134 strain was lab adapted to obtain streptomycin resistance. The obtained resistant mutant was grown in Stainer Scholte (SS) medium + 100 μg/ml streptomycin at 37 °C to reach an OD_600nm_ of 2.0. Genomic DNA was extracted using a QIAmp DNA minikit (Qiagen) and a sequencing library was prepared using Ion Shear Plus and Ion Plus Fragment kits (Life Technologies). The library was sequenced on an Ion Torrent PGM using a 314® chip (Life Technologies). The raw reads are available in SRA:SRR4019415 [28]. Cutadapt [10] (options -m 50 -q 20) was used to filter and trim the bad quality bases. A total of 623,304 reads were obtained after the quality trimming process.

The five Ion Torrent runs from the German outbreak were downloaded from [33] (Run 1-5), representing a total of 629,368 reads with a mean length of 105 bp and with qualities varying from Q28 to Q10, resulting in a mean theoretical depth of 10×. No pre-processing steps were performed. Illumina reads for *E. coli* 2009-2050 [50] and 2009-2071 [51] strains were extracted, respectively, from SRR647664 and SRR647666 obtained from the SRA databank. Reads sampling was performed with fastq-sample [52] to obtain a mean depth of 40 × .

### Evaluation of the annotations

In the 2011 German outbreak study, the annotation used as ground truth was the BROAD Institute annotation of the last assembly that included 5164 genomic genes and 189 genes from three plasmids. The gene sequences were downloaded from [53]. BG7 could not be run because of bugs in the program. The results corresponding to the annotation of the 3057 contigs and including 6190 genes were downloaded from [54]. The RAST web interface was used with the 3057 contigs and the reference organism selected was *E. coli* (taxid:562). Prokka was used with default parameters and the 3057 contigs.

To compare the annotation results, an in-house script was used to BLAST the BROAD genes against the CDSs predicted by each tool. Once a sequence was identified as present, it was removed from the list in order to count exactly the correct number of similar gene sequences. A predicted CDS was considered as a true positive if it has a significant hit in the BROAD gene sequences, as a false positive if it has no BLAST hit, and as a false negative if no BROAD reference gene had a sequence similar to that predicted by the annotation tool. Precision and recall values were computed as follows: precision = TP/(TP + FP) and recall = TP/(TP + FN)*.* The F-measure combines the precision and recall values and was computed as: F-measure = 2*(Precision*Recall)/(Precision + Recall)*.*

### Evaluation of the predicted antibiotic susceptibility profile

At the beginning of the outbreak, the Robert Koch Institute provided the microbial characterization, including the clinically important antibiotic susceptibility profile [55]. These data were used to evaluate the antibiotic susceptibility predictions obtained with MICRA and two others methods. In [37] 364 contigs from a later assembly (NCBI:AFOB01) were BLAST against DrugBank and ARDB. ResFinder [38], which is able to take raw reads as input, here the five Ion Torrent runs, assembled the reads into contigs and searched for susceptibility and resistance genes.

### Parameter settings

All the result files generated with MICRA and the complete description of parameters used in each experiment can be found in Additional file 1: part 5 and at [24]. IonGAP [25] was run with the sequencing DH10B FASTQ file and NCBI:CP000948 as reference genome in the formal context and with the five Ion Torrent runs and the *E. coli 55989* genome (NCBI :NC_011748) as reference in the clinical context. MIRA [16] assembler was used with its default parameters optimized for Ion Torrent reads (job = denovo,genome,accurate ; technology = iontor) and 24 threads. QUAST [26] was used with its default parameters.

## Availability and requirements

Poject name: MICRA

Project home page: http://www.pegase-biosciences.com/MICRA

Source code: https://github.com/caboche/MICRA

Archived version: https://zenodo.org/record/1045801

Operating system(s): Platform independent

Programming language: Java

Other requirements: none

License: GNU GPLv3

## Additional file

Additional file 1: Supplementary figures, notes, and tables. (PDF 3297 kb)

## References

[1] Fraser-Liggett CM (2005). Insights on biology and evolution from microbial genome sequencing. Genome Res.

[2] Wilson MR, Naccache SN, Samayoa E, Biagtan M, Bashir H, Yu G (2014). Actionable diagnosis of neuroleptospirosis by next-generation sequencing. N Engl J Med.

[3] Caboche S, Audebert C, Hot D (2014). High-throughput sequencing, a versatile weapon to support genome-based diagnosis in infectious diseases: applications to clinical bacteriology. Pathog.

[4] Robinson ER, Walker TM, Pallen MJ (2013). Genomics and outbreak investigation: from sequence to consequence. Genome Med.

[5] Goldberg B, Sichtig H, Geyer C, Ledeboer N, Weinstock GM (2015). Making the leap from research laboratory to clinic: challenges and opportunities for next-generation sequencing in infectious disease diagnostics. MBio.

[6] Fricke WF, Rasko D (2014). Bacterial genome sequencing in the clinic: bioinformatic challenges and solutions. Nat Rev Genet.

[7] Caboche S, Audebert C, Lemoine Y, Hot D (2014). Comparison of mapping algorithms used in high-throughput sequencing: application to Ion Torrent data. BMC Genomics.

[8] Caboche S, Even G, Loywick A, Audebert C, Hot D. MICRA web interface. PEGASE. 2017. http://www.pegase-biosciences.com/MICRA. 10.1186/s13059-017-1367-z PMC5738152 29258574

[9] Andrews S. FastQC. www.bioinformatics.babraham.ac.uk/projects/fastqc/.

[10] Martin M (2011). Cutadapt removes adapter sequences from high-throughput sequencing reads. EMBnet J.

[11] Zaharia M, Bolosky WJ, Curtis K, Fox A, Patterson D, Shenker S (2011). Faster and more accurate sequence alignment with SNAP.

[12] David M, Dzamba M, Lister D, Ilie L, Brudno M (2011). SHRiMP2: sensitive yet practical short read mapping. Bioinformatics.

[13] Langmead B, Salzberg SL (2012). Fast gapped-read alignment with Bowtie 2. Nat Methods.

[14] Stothard P, Wishart DS. Circular genome visualization and exploration using CGView. Bioinformatics. 2005;21:537–9. 10.1093/bioinformatics/bti054 15479716

[15] Morgulis A, Gertz EM, Schäffer AA, Agarwala R (2006). A fast and symmetric DUST implementation to mask low-complexity DNA sequences. J Comput Biol.

[16] Chevreux B. MIRA: an automated genome and EST assembler. Duisbg Heidelb. 2005;1–161. https://www.scienceopen.com/document?vid=bc89e336-6dbb-4369-853b-3cb2fdc015b2.

[17] Bankevich A, Nurk S, Antipov D, Gurevich AA, Dvorkin M, Kulikov AS, et al. SPAdes: a new genome assembly algorithm and its applications to single-cell sequencing. J Comput Biol. 2012;19:455–77. 10.1089/cmb.2012.0021 PMC3342519 22506599

[18] Gillespie JJ, Wattam AR, Cammer SA, Gabbard JL, Shukla MP, Dalay O (2011). Patric: The comprehensive bacterial bioinformatics resource with a focus on human pathogenic species. Infect Immun.

[19] Wishart DS, Knox C, Guo AC, Shrivastava S, Hassanali M, Stothard P (2006). DrugBank: a comprehensive resource for in silico drug discovery and exploration. Nucleic Acids Res.

[20] Liu B, Pop M (2009). ARDB--Antibiotic Resistance Genes Database. Nucleic Acids Res.

[21] Caboche S, Even G, Loywick A, Audebert C, Hot D. MICRA documentation. PEGASE. 2017. http://www.pegase-biosciences.com/MICRA/help.php. 10.1186/s13059-017-1367-z PMC5738152 29258574

[22] Huang W, Li L, Myers JR, Marth GT (2012). ART: a next-generation sequencing read simulator. Bioinformatics.

[23] Garrison E, Marth G. Haplotype-based variant detection from short-read sequencing. arXiv Prepr. arXiv1207.3907. 2012;9. http://arxiv.org/abs/1207.3907.

[24] Caboche S, Even G, Loywick A, Audebert C, Hot D. MICRA data. PEGASE. 2017. http://www.pegase-biosciences.com/MICRA/data.html. 10.1186/s13059-017-1367-z PMC5738152 29258574

[25] Baez-Ortega A, Lorenzo-Diaz F, Hernandez M, Gonzalez-Vila CI, Roda-Garcia JL, Colebrook M (2015). IonGAP: integrative bacterial genome analysis for Ion Torrent sequence data. Bioinformatics.

[26] Gurevich A, Saveliev V, Vyahhi N, Tesler G (2013). QUAST: quality assessment tool for genome assemblies. Bioinformatics.

[27] Weber C, Boursaux-Eude C, Coralie G, Caro V, Guiso N (2001). Polymorphism of Bordetella pertussis isolates circulating for the last 10 years in France, where a single effective whole-cell vaccine has been used for more than 30 years. J Clin Microbiol.

[28] Caboche S, Even G, Loywick A, Audebert C, Hot D. SRA:SRR4019415. Sequence Read Archive; 2016. http://www.ncbi.nlm.nih.gov/sra.

[29] Caro V, Hot D, Guigon G, Hubans C, Arrivé M, Soubigou G (2006). Temporal analysis of French Bordetella pertussis isolates by comparative whole-genome hybridization. Microbes Infect.

[30] Mielcarek N, Debrie AS, Raze D, Quatannens J, Engle J, Goldman WE (2006). Attenuated Bordetella pertussis: new live vaccines for intranasal immunisation. Vaccine.

[31] Scheutz F, Nielsen EM, Frimodt-Møller J, Boisen N, Morabito S, Tozzoli R, et al. Characteristics of the enteroaggregative Shiga toxin/verotoxin-producing Escherichia coli O104:H4 strain causing the outbreak of haemolytic uraemic syndrome in Germany, May to June 2011. Euro Surveill. 2011;16. https://www.ncbi.nlm.nih.gov/pubmed/?term=Characteristics+of+the+enteroaggregative+Shiga+toxin%2Fverotoxin-producing+Escherichia+coli+O104%3AH4+strain+causing+the+outbreak+of+haemolytic+uraemic+syndrome+in+Germany%2C+May+to+June+2011. 10.2807/ese.16.24.19889-en 21699770

[32] Rohde H, Qin J, Cui Y, Li D, Loman NJ, Hentschke M (2011). Open-source genomic analysis of Shiga-toxin-producing E. coli O104:H4. N Engl J Med.

[33] BGI. Ion Torrent reads for E. coli O104:H4 2011. ftp://ftp.genomics.org.cn/pub/Ecoli_TY-2482/.

[34] Pareja-Tobes P, Manrique M, Pareja-Tobes E, Pareja E, Tobes R (2012). BG7: a new approach for bacterial genome annotation designed for next generation sequencing data. PLoS ONE.

[35] Aziz RK, Bartels D, Best AA, DeJongh M, Disz T, Edwards RA (2008). The RAST Server: rapid annotations using subsystems technology. BMC Genomics.

[36] Seemann T (2014). Prokka: rapid prokaryotic genome annotation. Bioinformatics.

[37] Kuznetsov V, Lee HK, Maurer-Stroh S, Molnár MJ, Pongor S, Eisenhaber B, et al. How bioinformatics influences health informatics: usage of biomolecular sequences, expression profiles and automated microscopic image analyses for clinical needs and public health. Heal Inf Sci Syst. 2012;1:2. https://www.ncbi.nlm.nih.gov/pubmed?term=how%20bioinformatics%20influences%20health%20informatics%20usage%20of%20biomolecular%20sequences,%20expression%20profiles%20and%20automated%20microscopic%20im 10.1186/2047-2501-1-2 PMC4336111 25825654

[38] Zankari E, Hasman H, Cosentino S, Vestergaard M, Rasmussen S, Lund O (2012). Identification of acquired antimicrobial resistance genes. J Antimicrob Chemother.

[39] Ahmed SA, Awosika J, Baldwin C, Bishop-Lilly KA, Biswas B, Broomall S (2012). Genomic comparison of Escherichia coli O104:H4 isolates from 2009 and 2011 reveals plasmid, and prophage heterogeneity, including Shiga toxin encoding phage stx2. PLoS ONE.

[40] Power RA, Parkhill J, de Oliveira T (2016). Microbial genome-wide association studies: lessons from human GWAS. Nat Rev Genet..

[41] Caboche S. MICRA source code. GitHub; 2017. https://github.com/caboche/MICRA.

[42] Caboche S (2017). MICRA source code.

[43] NCBI. FTP bactarial genomes. NCBI. ftp://ftp.ncbi.nlm.nih.gov/genomes/archive/old_refseq/Bacteria/summary.txt.

[44] NCBI. FTP bacterial plasmids. NCBI. ftp://ftp.ncbi.nlm.nih.gov/genomes/archive/old_refseq/Plasmids/Plasmids.ids.

[45] Fu L, Niu B, Zhu Z, Wu S, Li W (2012). CD-HIT: accelerated for clustering the next-generation sequencing data. Bioinformatics.

[46] McArthur AG, Waglechner N, Nizam F, Yan A, Azad MA, Baylay AJ (2013). The comprehensive antibiotic resistance database. Antimicrob Agents Chemother..

[47] Altschul SF, Madden TL, Schäffer AA, Zhang J, Zhang Z, Miller W (1997). Gapped BLAST and PSI-BLAST: a new generation of protein database search programs. Nucleic Acids Res.

[48] McKay S GD. bp_genbank2gff3.pl. GitHub; 2014. https://github.com/bioperl/bioperl-live/blob/master/scripts/Bio-DB-GFF/bp_genbank2gff3.pl.

[49] Chen H, Boutros PC (2011). VennDiagram: a package for the generation of highly-customizable Venn and Euler diagrams in R. BMC Bioinformatics.

[50] NCBI. SRR647664: reads for E. coli 2009-2050. SRA. http://www.ncbi.nlm.nih.gov/sra.

[51] NCBI. SRR647666: reads for E. coli 2009-2071. SRA. http://www.ncbi.nlm.nih.gov/sra.

[52] DC Jones. FASTQ-SAMPLE. https://homes.cs.washington.edu/~dcjones/fastq-tools/fastq-sample.html.

[53] Broad Institute. Escherichia coli O104:H4 sequencing project. http://www.broadinstitute.org/annotation/genome/Ecoli_O104_H4/MultiDownloads.html.

[54] BGI. Annotation of E. coli O104:H4. GitHub. https://github.com/ehec-outbreak-crowdsourced/BGI-data-analysis/tree/master/strains/TY2482/seqProject/BGI/annotations/era7bioinformatics.

[55] Robert Koch Institute. antibiotic susceptibility profile for E. coli O104:H4. http://www.rki.de/EN/Content/infections/epidemiology/outbreaks/EHEC_O104/ehec_O104_inhalt_en.html.

