# Supplementary material for: MICRA: an automatic pipeline for fast characterization of microbial genomes from high-throughput sequencing data
Source: Genome Biol. 2017 Dec 19;18:233. doi: 10.1186/s13059-017-1367-z (PMC5738152; doi:10.1186/s13059-017-1367-z)
Supplement: Additional file 1: — Supplementary figures, notes, and tables. (PDF 3297 kb) [file 13059_2017_1367_MOESM1_ESM.pdf]

Supplementary information

This file contains supplementary information for the paper 'An automatic pipeline for fast characterization of microbial genomes from high-throughput sequencing data'. The first part is a validation study for the selection of reference sequences. Complementary results from the study of P134S strain of *Bordetella pertussis* are given in the second part. The third part contains additional evaluation results of the study concerning the 2011 German outbreak. The fourth part contains a complete case studies on *Staphylococcus aureus* and *Clostridium autoethanogenum*. Finally, a last part lists the parameters used in the different analysis performed for this study.

1- Validation study for the selection of reference sequences

Selection of reference sequences is a critical step in MICRA. This step has to be reliable and efficient as the quality of results is highly dependent of the selected sequences. Reference sequences are selected based on the number of BLAST hits return from a subset of N reads randomly drawn against the MICRA databases of genome or plasmid sequences. To validate our method, the selection module was run 50 times and the list of selected sequences (5 sequences for genome and 10 for plasmids) were compared to the sequences identified from a more exhaustive search using contigs from a complete *de novo* assembly instead of reads. We computed the size of intersection between the list of entries selected from *de novo* contigs and the list of entries selected directly from reads. Statistical tests were performed to evaluate if the results are different between the considered conditions (Wilcoxon test for two conditions and Kruskal Wallis test for more than two conditions). Figure S1 shows the results of this validation study for the MICRA selection of reference sequences.

We used all the datasets presented in the manuscript and supplementary material to evaluate the sequence selection step: *Staphylococcus aureus* Ilumina paired-end (PE) [Figure S1 A, B C and D] and Ion Torrent (IT) data [figure S1 E and F], *Clostridium autoethanogenum* PE [Figure S1 I and J] and IT [Figure S1 K] data, *Bordetella pertussis* Pillemer strain IT data [Figure S1 L], *Escherichia coli* O104:H4 IT data [Figure S1 G and H]. These datasets represented a goof variability in bacterial strains and also in sequencing technologies having different read sizes and error rates.

As MICRA only used the reads from the first file of Illumina PE datasets, we first compare the results produced from read file 1 and read file 2 with 1,000 reads for genome selection and 10,000 reads for plasmid selection (Figure S1 B, D and J). All the results showed that use of file 1 or file 2 did not produce different results (p-value=1 in B and results strictly identical in D and J).

In order to choose the better compromise between time efficiency and reliability, we tested for several numbers of reads used for selection: 100, 1,000 and 10,000 reads for genome selection and 1,000, 10,000 and 100,000 reads for plasmid selection. For *Staphylococcus aureus* PE dataset the MICRA list contains 3 or 4 genome sequences in common with the 5 sequences selected from contigs whatever the number of reads (Figure S1 A, p-value=0,81). The different sequences were very genetically closed and so did not impact the MICRA results. There were 4 common entries over 10 for plasmids (Figure S1 C, p-value=0.73). These 4 entries were the closest sequences for plasmids, the other being very distant from the true reference sequences and were then selected arbitrary with a very few number of reads (but did not impact the results since those plasmid sequences were filtered out by the next module of MICRA). Similar results were obtained with the IT dataset with 3 common genome sequences (Figure S1 E). However, the results for the plasmid selection were more variable depending on the number of reads. They stabilized with 10,000 and 100,000 reads leading to 6 common sequences (Figure S1 F). The conclusion can be drawn with the *Escherichia coli* O104:H4 IT dataset with 4 common genome sequences and 6 common plasmid sequences (Figure S1 G and H). For *Clostridium autoethanogenum* dataset only one genome in the MICRA database is really closed to the true reference sequence and this is the one identified by

both of the approaches. With the *Clostridium autoethanogenum* IT dataset 4 genome sequences were commonly identified by both approaches (Figure S1 K). For *Bordetella pertussis* Pillemer strain dataset MICRA identified exactly the same five genome sequences than the ones identified from *de novo* contigs whatever the number of reads (Figure S1 L). These results were due to the fact that few Bordetella genome sequences are available in database so they were always selected.

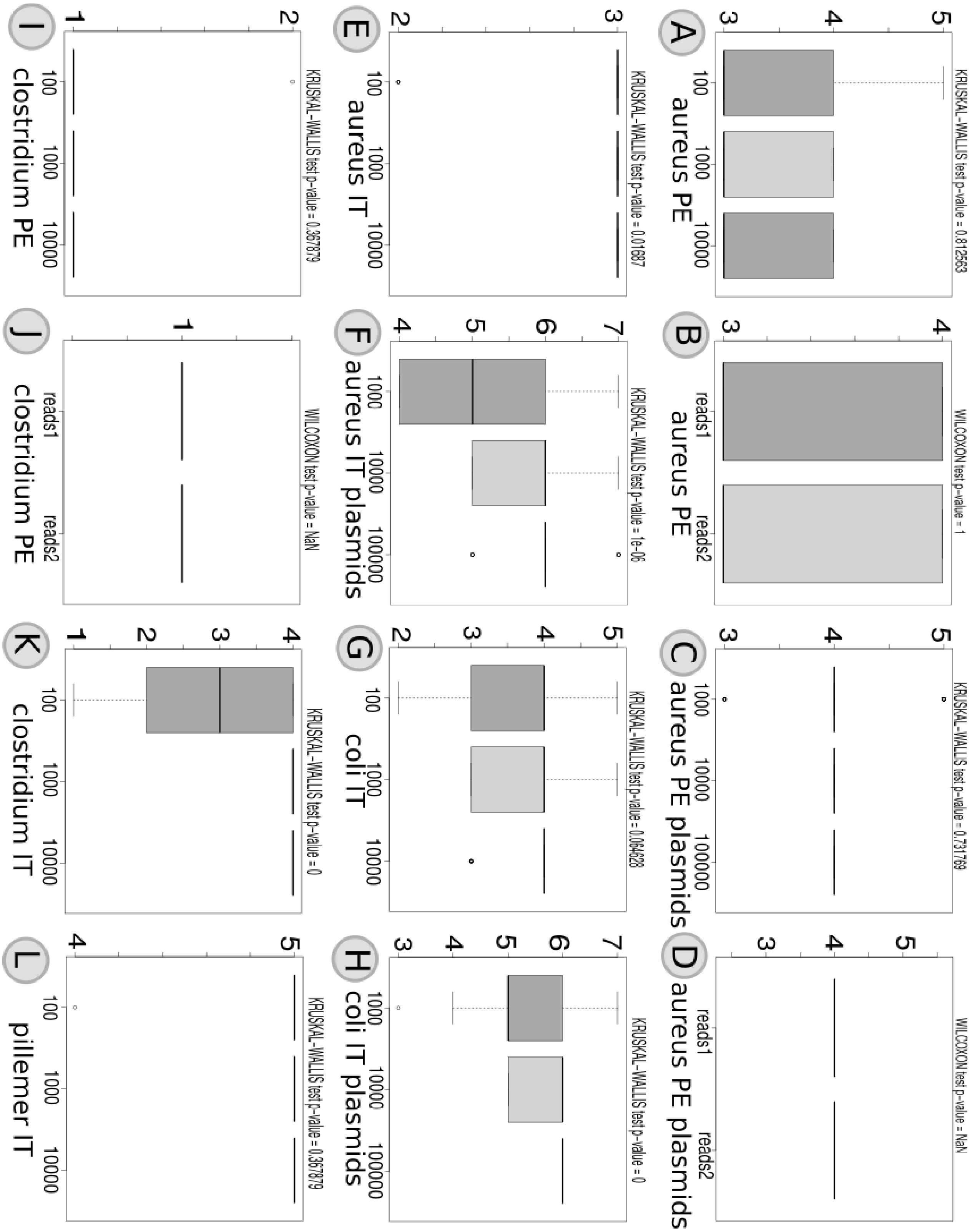

Figure S1: Validation study for the MICRA selection of reference sequences.

All the results showed that the results are stable for multiple runs in which the subset of reads was randomly selected (only small variations on sequence set more genetically distant from the real sequence) and that 1,000 reads and 10,000 reads are enough to obtained stable and reliable selection of reference sequences in a very short time (only several seconds). These values are used as default values in MICRA but the user can increase those numbers which will also increase the running-time. Finally, these results validated that the MICRA selection method is reliable and efficient leading to the identification of closest reference sequences.

2- Supplementary data for the study of Pillemer P134S strain of *Bordetella pertussis*

2-1 List of P134S closest reference genomes and comparison values as determined by MICRA first module

Bordetella pertussis Pillemer P134S genome was extracted from a log-phase stopped culture in Stainer Scholte medium at 37 °C. Genomic DNA was prepared using Ion Shear Plus and Ion Plus Fragment kits from Life technologies for a sequencing run on an Ion Torrent PGM (314-chip). Reads were quality-trimmed with cutadapt [1] and analyzed using MICRA. The first module produced the following statistics table (Table S1):

<table>
<tr><th>genome</th><th>size(bp)</th><th>coverage</th><th>MD</th><th>sd</th><th>%mapped</th><th>min,max</th><th>#SNVs</th><th>#SNVs_CDS</th><th>#SNVs_coding</th><th>#INDELs</th><th>#IND_CDS</th></tr>
<tr><td>B-petrii-strain-DSM-12804:NC_010170.1</td><td>5287950</td><td>15.2857</td><td>1.90</td><td>5.36</td><td>14.54</td><td>0|79</td><td>72566</td><td>70919</td><td>18220</td><td>1920</td><td>1539</td></tr>
<tr><td>B-parapertussis-strain-12822:NC_002928.3</td><td>4773551</td><td>70.6566</td><td>12.34</td><td>10.51</td><td>84.57</td><td>0|96</td><td>28901</td><td>23044</td><td>7443</td><td>692</td><td>304</td></tr>
<tr><td>B-pertussis-Tohama-I:NC_002929.2</td><td>4086189</td><td>88.5352</td><td>16.85</td><td>48.31</td><td>98.78</td><td>0|3809</td><td>165</td><td>105</td><td>63</td><td>41</td><td>30</td></tr>
<tr><td>B-bronchiseptica-strain-RB50:NC_002927.3</td><td>5339179</td><td>65.5193</td><td>11.35</td><td>10.53</td><td>87.05</td><td>0|72</td><td>28743</td><td>24522</td><td>7299</td><td>673</td><td>338</td></tr>
<tr><td>Achromo-xylosoxidans-A8:NC_014640.1</td><td>7013095</td><td>12.6998</td><td>1.60</td><td>4.97</td><td>16.27</td><td>0|69</td><td>79773</td><td>78218</td><td>21377</td><td>2038</td><td>1668</td></tr>
</table>

Table S1 : Mapping statistics produced by MICRA in the fast mapping step
bp = base pair, sd = standard deviation of sequencing depth, coverage= percentage of sequence coverage, % mapped = percentage of mapped read on the considered genome, min|max = minimal, maximal sequencing depth, # = number of, “CDS” = within CDS, “coding” = coding variation.

Four genomes were determined by MICRA as close enough to be considered as reference genomes: *Bordetella pertussis* strain Tohama I, *Bordetella bronchiseptica* strain RB50, *Bordetella parapertussis* strain 12822, *Bordetella petrii* strain DSM12804 and *Achromobacter xylosoxidans* strain A8. The closest genome is *Bordetella pertussis* strain Tohama I with more than 98% of its genome covered and the smallest numbers of SNVs and INDELs.
This result table provides a quick and integrated comparison of the selected genomes to the analyzed sequence. Variations in term of coding and non-coding SNVs and INDELs are reported and easily comparable between references.

2-2 List of variations between *Bordetella pertussis* P134S and Tohama I strains

The list of variations is available on http://www.pegase-biosciences.com/MICRA/data.html. For each variation the table returns the variation base position in the reference genome (column “ref.position”), the type of variation SNV or INDEL (column “type”), the expected base on the reference genome (column “ref. base”), the base predicted by the MICRA variant caller (column “variant”), the count of variant base (column “counts”), the frequency of variant base (column “frequency”), the total sequencing depth (column “depth”), the name and function or predicted function of impacted CDS, if any (column “CDS”), the amino acid modification, if any, with the position within the CDS between brackets (column “AA change”). The reliability of variations can be assessed by the sequencing depth and frequencies of observed variant bases. In this example some of the variation positions are not deeply sequenced and are therefore to be considered with caution.

Several variations are detected in important characterized genes which might be of impact

on the general fitting and physiology of the studied strain. For example, one can mention coding variation in the *bvgS* gene which codes for the sensor protein of the main virulence-regulator two-component system of the bacteria [2,3]. Another coding variation lays in *ptxB* gene, the subunit 2 of pertussis toxin which is one of the main toxin of the pathogen. Two variations are also predicted in *fimC* (INDEL) and in *fimD* (coding SNV) which are the outer membrane protein and the adhesin protein, respectively, of fimbriae secretory system. These 3 variations were validated by Sanger sequencing (see part 1-3). Other coding and non-coding variations could also be of importance as they occur in strategic genes such as lipoproteins, transporters, transcriptional regulators and metabolic enzymes.

2-3 SNVs and INDELs validation by Sanger sequencing

For each of the five tested variations, a forward and a reverse PCR primers were designed around the position of the variation. These primers were used to PCR amplify the surrounding regions of the variations and they were then used in Sanger sequencing reactions. The chromatograms from forward and reverse strand sequences are presented below. In each panel below, the analyzed sequence region is indicated with the used sequencing primers highlighted in yellow and grey. The variation (SNV or INDEL) are localized by a red highlight. Part of the Sanger sequencing chromatograms of forward and reverse strand containing the variation is shown. The variation position is indicated on the chromatogram by a red arrow.

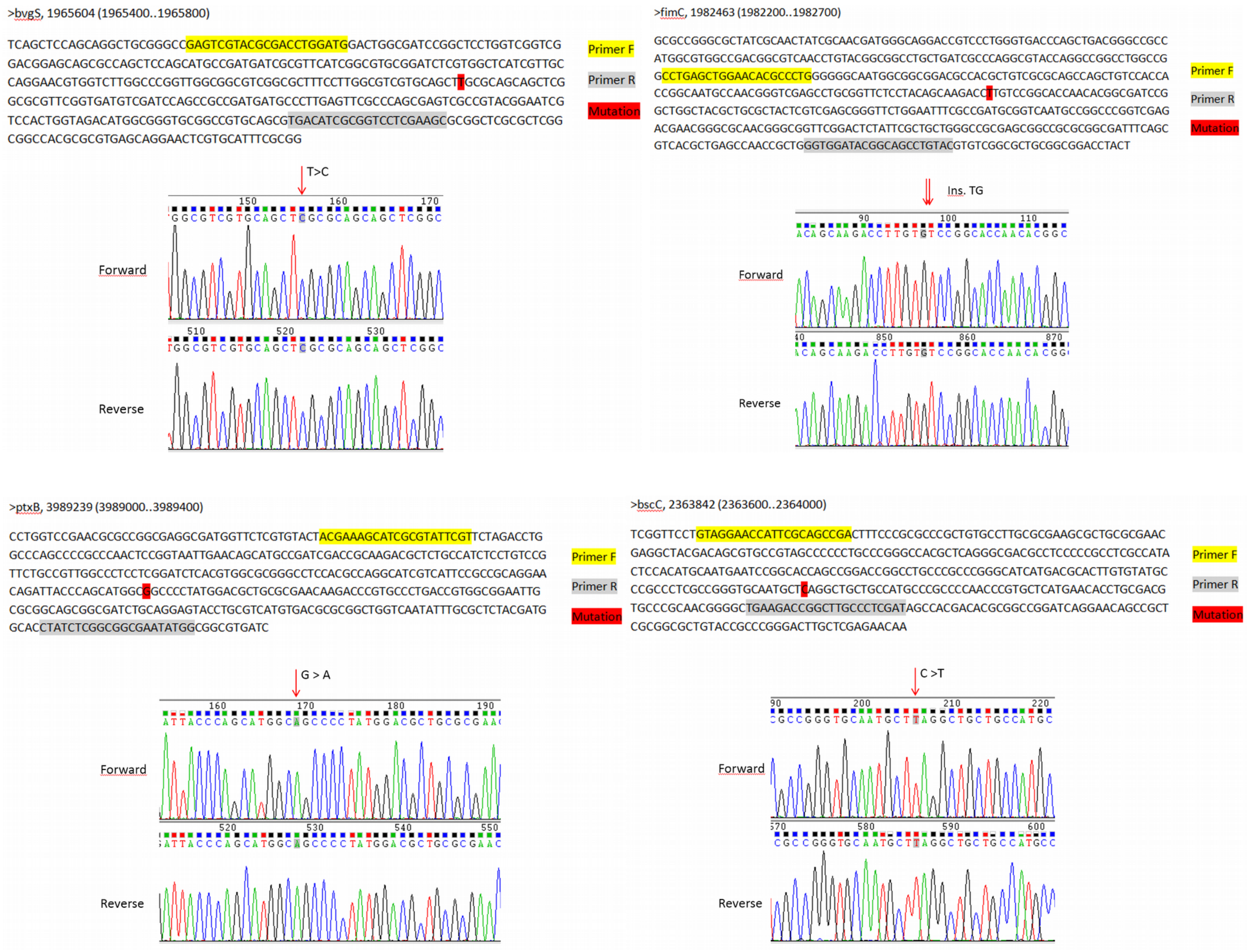

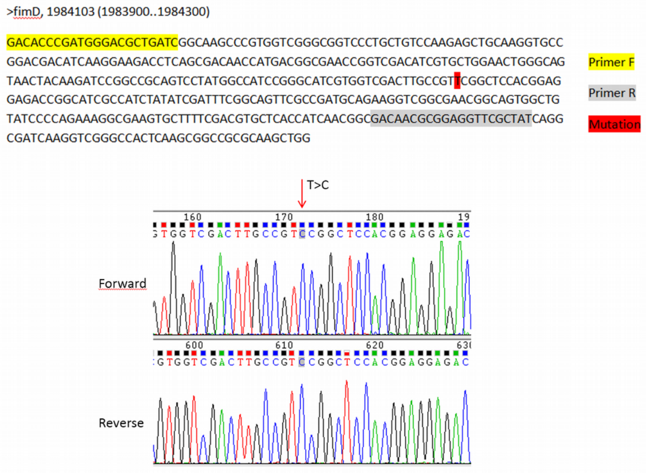

2-4 Validation of additional regions within P134S genome compare to Tohama I genome

Four additional regions were detected by MICRA in the P134S genome compared to Tohama I. These regions were detected to be highly similar to four clusters of genes presents in *Bordetella bronchiseptica* RB50 genome: BB0534 to BB0541, BB0916 to BB0921, BB1141 to BB1158 and BB4880 to BB4888. A PCR was designed in one gene of each of these 4 clusters (BB0538, BB0916, BB1148 and BB4884) using the sequence of *Bordetella bronchiseptica* RB50 (NC_002927) as template. A PCR amplification was detected for P134S whereas the same PCR conditions applied to Tohama I genome does not give any amplification (see PCR bands detection on agarose gel below) confirming the presence on these genes in the P134S genome and their absence from TohamaI strain.

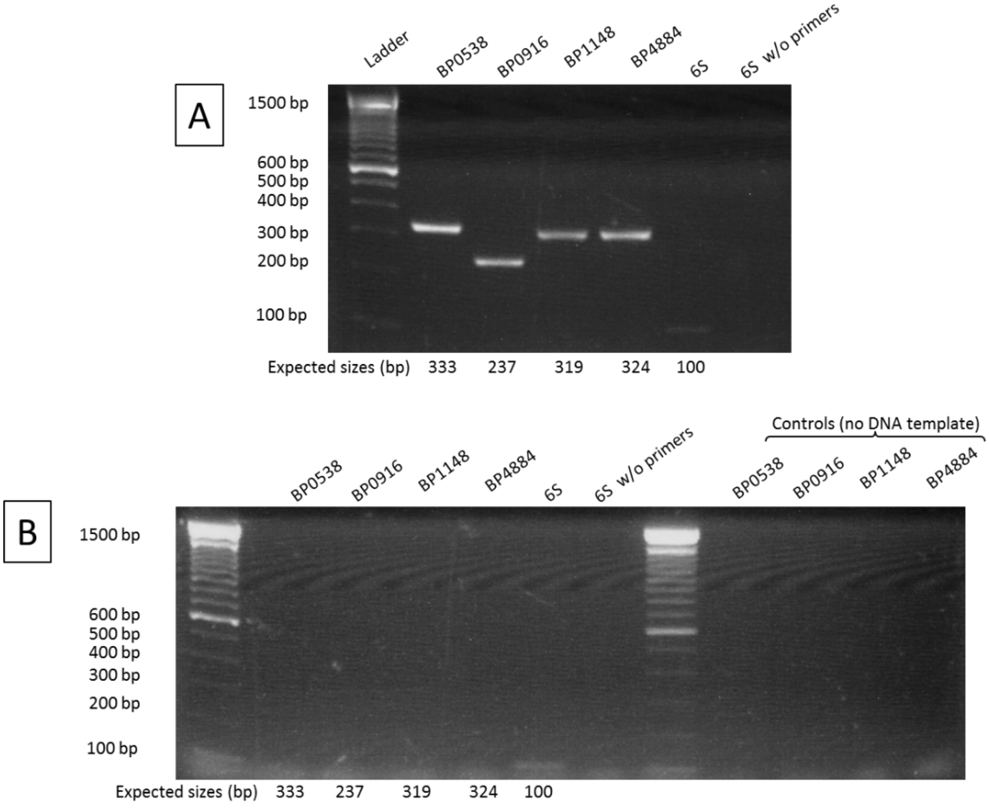

Supplementary part2-4: PCR detection of additional regions within Pillemer genome compare to Tohama I genome. Gene names targeted by PCR primers are indicated above the gel. The 6S gene is used as positive control as it is present in the 2 strains. Expected sizes are indicated below the gels. (A): PCR products obtained using Tohama I strain as template. (B): PCR products obtained using Pillemer strain as template. The right part of the gel are negative controls without DNA template.

3 – Supplementary data for the 2011 German outbreak caused by Escherichia coli O104:H4

3-1 MICRA used with the TY2482 sequences

MICRA was used with the TY2482 chromosome and the three plasmid sequences (downloaded from ftp://ftp.genomics.org.cn/pub/Ecoli_TY-2482/) to evaluate the possible power of the analysis, independently of the choice of the reference sequences given in input. The results are shown in table S2.

	size	Coverage (%)	mean depth	consensus	#variant calls	#CDS	absent	present	LC	partial	divergent
chromosome	5278900	99.0489	10.64	629	45 (24+21)	5164	0	5130	1	26	7
pESBL	88695	98.5952	11.67	13	3 (3+0)	100	0	98	0	2	0
pAA	75330	73.6002	9.59	36	0	87	19	56	0	3	9
PG	1549	100.0	410.01	1	0	2	0	2	0	0	0

Table S2: Results obtained with MICRA considering the TY2482 chromosome and plasmid sequences
“Size” column gives the reference sequence size, the percentage of the covered reference sequence is given in the third column. The mean sequencing depth is given in column 4. The “consensus” column shows the number of consensus sequences generated from the mapping. The number of variant calls is given in the next columns, numbers in parenthesis are respectively the number of SNVs and the number of INDELs. The number of annotated CDS on the reference sequence is represented in the “#CDSs” column. The 5 last columns give the respective number of CDSs returned by MICRA as absent, present, low complexity (LC) region, partially covered (partial) and divergent.

The chromosome sequence is covered at more than 99%. Despite the poor quality and the low throughput of the sequencing, only 45 SNVs are called. The major part of the annotated CDSs were identified as present. The plasmid pESBL is covered at more than 98% and 98% of annotated CDSs were categorized as present. Three false-positive variants were predicted. For the plasmid pAA the surprising result was the low percentage of coverage around 73%. Blasting the pAA sequence against the chromosome sequence showed a similar region representing 26% of the pAA sequence. The reads corresponding to this similar region have been probably already mapped onto the chromosome sequence explaining the lower percentage of coverage of the pAA plasmid. The number of reads in the corresponding genomic region showed an increased depth confirming the hypothesis (data not shown). The pG plasmid sequence was entirely covered and no variant was called. The mean depth observed for the chromosome and large plasmids were close to the theoretical sequencing depth (around 10X) whereas for pG plasmid, a mean depth of around 400X was observed, meaning that the copy number of this plasmid should be higher. This result was consistent with results from a previous study [4]. At the end of the iterative mapping step, 52,312 reads were still unmapped and were then *de novo* assembled. However, no contig with a size greater than 500 nucleotides were obtained meaning that the remaining reads were probably of very low quality (which was confirmed by FastQC [5]).

3-2 Comparison of MICRA with *de novo* assembly based approaches

We compared the MICRA results with those obtained with *de novo* based approaches. During the outbreak, *de novo* assembly was rapidly performed from the 5 IonTorrent runs with the MIRA assembler and produced 3,057 contigs (downloaded from https://github.com/ehec-outbreak-crowdsourced/BGI-data-analysis/wiki/Assemblies). Around thirty minutes were necessary to complete this assembly. QUAST was used to compare the results of MICRA run with the TY2482 sequences (MICRA_ref), MICRA run in automatic way (MICRA), the assembly produced during the outbreak (3057 contigs), MIRA and IonGAP with the 5 Ion torrent runs (Table S3).

	#contigs >=500	N50	Fraction (%)	#genes	#misass.	#Ns	#mm	#indels
				chromosome				
MICRA_ref	542	15966	98.808	4711+431 part	0	11268	17	20 (20+0)
MICRA	723	13904	95.79	4330+590 part	56	11182	2353	294 (264+30)
3057 contigs	1914	3957	94.266	3535+1440 part	27	776	1741	7696 (7621+75)
MIRA	2387	2822	91.989	3088+1851 part	22	515	1363	5655 (5595+60)
IonGAP	2531	2542	91.04	2889+2033 part	24	212	2081	4998 (4957+41)
				pESBL				
MICRA_ref	12	10735	98.343	89+10 part	0	66	3	0
MICRA	13	10498	98.108	84+13 part	0	68	5	9 (6+3)
3057 contigs	42	2974	96.781	71+28 part	3	19	17	128 (127+1)
MIRA	47	2974	96.009	62+37 part	2	5	13	116 (113+3)
IonGAP	41	3054	94.91	63+34 part	1	7	11	100 (98+2)
				pAA				
MICRA_ref	21	4823	65.813	47+11 part	0	76	0	0
MICRA	50	12462	72.525	48+19 part	0	551	56	50 (48+2)
3057 contigs	48	1368	74.608	45+23 part	0	17	100	76 (73+3)
MIRA	46	1693	72.877	44+24 part	1	10	74	58 (57+1)
IonGAP	44	2370	80.753	50+26 part	1	4	96	54 (52+2)
				pG				
MICRA_ref	1	1549	100	2+0 part	0	0	0	0
MICRA	1	1549	100	1+1 part	0	0	3	0
3057 contigs	1	1819	100	2+0 part	0	0	0	0
MIRA	1	1766	100	2+0 part	0	0	0	2 (2+0)
IonGAP	1	1777	100	1+1 part	0	0	0	2 (2+0)

Table S3: Evaluation with QUAST of assemblies obtained with MICRA, IonGAP and MIRA

The number of contigs (*de novo* contigs + consensus sequences from mapping) produced by MICRA is significantly lower and the N50 is greater compared with *de novo* based approaches. The genome fraction is similar between the two approaches. A surprising result is obtained for the pAA plasmid which is covered at only 65% with MICRA using the TY2482 sequences whereas it is covered between 72 and 80% with the other approaches. This difference is mainly explained by the similar region between pAA and the chromosome sequences that deplete the reads using MICRA with the TY2482 sequences, whereas using MICRA in automatic way select a genome sequences for which this shared region is less similar. An interesting result is the length of the contig for the pG plasmid; pG is a small plasmid of 1549 bp, contrary to MICRA the *de novo* based approaches lead to a contig larger than the plasmid size meaning that there is an error during the *de novo* assembly process. The number of miss-assemblies is greater for the MICRA assembly with mainly relocations due to the difference of gene order between the sequenced genome and the reference genomes. The number of ambiguous bases N is 10 to 50-fold greater in the sequences produced by MICRA that is explained by the poor quality of the sequencing resulting in low coverage regions which are encoded by N symbols in the MICRA pipeline. MICRA induced more mismatches for the genome sequence than the *de novo* approaches but much less indels. Indels are the main errors made by Ion torrent technology and are taken into account into MICRA explaining the lower number of indels in sequences generated by MICRA.

3-3 Analysis of the 2009 strains

Two *Escherichia coli O104:H4* isolates close to TY2482 were sequenced and studied in 2011 [6]: 2009EL-2050 and 2009EL-2071. A combination of single and paired-end Illumina reads, 454 technologies and additional finishing reactions were used to produce the complete genome sequences. We used a subset of the single Illumina reads to achieve a theoretical depth of 40X, *i.e.*

2,120,000 reads of 100 bp length.

Firstly, only the pre-process module of MICRA was run with the local version of the database in which the sequences released after 2011 were discarded. Four out of the 5 reference genomes identified were identical and so we decided to use the same 5 reference genomes than the ones used in the study of the TY2482 isolate, allowing us to easily compare the variant calls and annotations between all the three strains with the MICRA-comparison tool. On the other hand, the plasmids could be specific of each isolate and the 10 automatically selected plasmids were kept for each strain (All the lists of reference sequences are available at http://www.pegase-biosciences.com/MICRA/data.html).

For the 2009EL-2050 strain, the closest reference genome is the genome of *Escherichia coli* 55989 (CU928145.2) covered at more than 95% with a mean depth of 34X. MICRA identified two plasmids: the small plasmid pCE10D of the *Escherichia coli O7K1* strain (CP003038.1), covered at 99.8% with a mean depth of 559X, corresponding to the pG plasmid and a new plasmid close to the *Escherichia coli LF82* plasmid (CU638872.1), covered at 74% with a mean depth of 20X. This last plasmid is specific to the 2009EL-2050 strain [6]. The plasmid close to pAA was not identified for the same reason as mentioned above. The closest reference genome for the 2009EL-2071 strain was also the 55989 strain, covered at 95% with a mean depth of 34X. Only the small pCE10D plasmid from the *Escherichia coli O7K1* strain was identified (covered at 99.7% with a mean depth of 805X).

4- Additional application examples

In order to confirm the performances of MICRA, we performed two additional complete case studies with *Staphylococcus aureus* and *Clostridium autoethanogenum*.

4-1 Study of *Staphylococcus aureus* data

To assess MICRA performances, we considered the *Staphylococcus aureus* strain (Terrabacteria group; Firmicutes; Bacilli; Bacillales; Staphylococcaceae; Staphylococcus). This is a standard strain used for technology validation as it exhibits a low GC percentage (around 33%). Illumina and Ion torrent data were used for this evaluation.

Paired-end Illumina sequencing data

The *Staphylococcus aureus* sequence Type 239 (TW20) strain, a multi-resistant strain which has emerged recently and which is highly transmissible, was completely described in [7]. It contains one chromosome (TW20 NCBI: NC_017331.1) and two plasmids: pTW20_1 (NCBI: NC_017352.1) and pTW20_2 (NCBI: NC_017332.1). The dataset (SRA : ERR1274626) contains 518,882 read pairs of 130bp with a quality higher than Q32.

MICRA was run with and without reference sequence. Without reference sequence, MICRA identified the *Staphylococcus aureus subsp. Aureus* NCTC 8325 genome (NCBI:NC_007795.1) as the closest reference sequence.

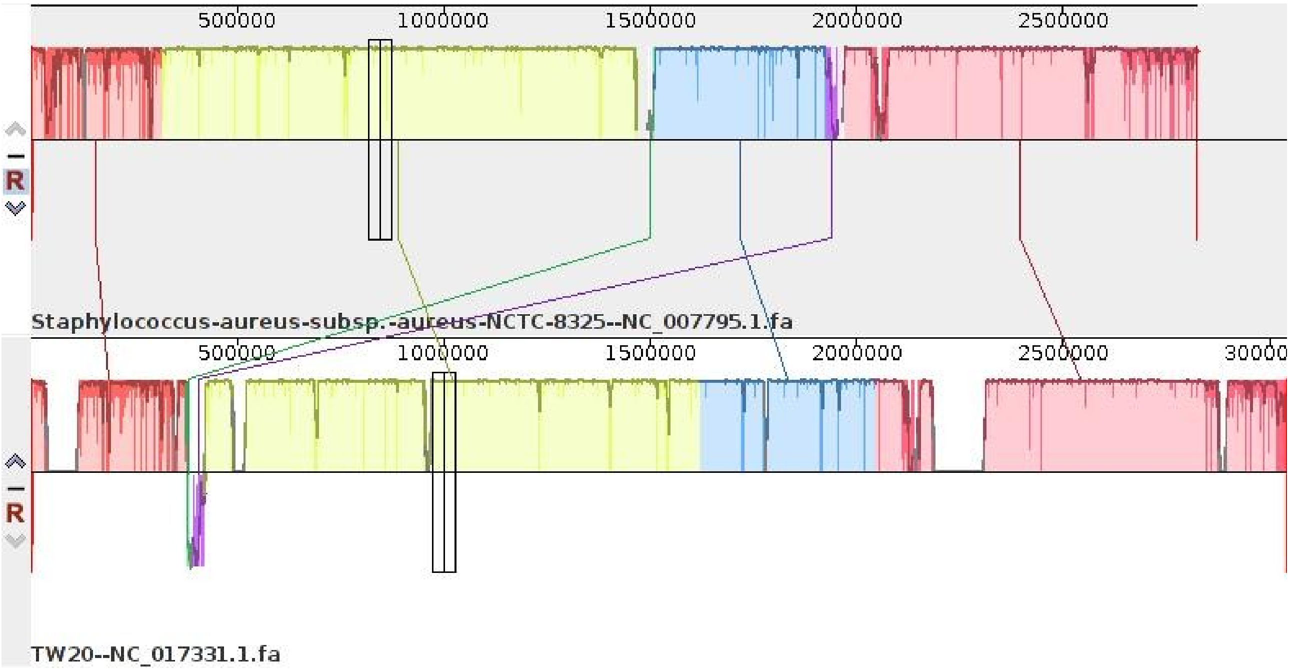

Figure S2: Comparison of TW20 and NTC8325 genomes using MAUVE

Figure S2 shows that the NTC8325 genome selected by MICRA presents regions homologous to TW20 genome sequence (around 95% is similar to the TW20 genome). However, the region organization is different and the TW20 genome contains additional specific regions. In MICRA, this genome sequence was covered at 94.26% with more than 78% of read pairs mapped resulting in a mean depth of 37X. After the iterative mapping step 94,655 pairs of reads were still unmapped and used in *de novo* assembly step producing 275 contigs greater than 500bp. The MICRA resulting sequences were compared to those obtained from a *de novo* assembly using SPAdes followed by the contig annotation using PROKKA. Table S4 shows the assembly comparison obtained with QUAST.

	#contigs	N50	% genome	# genes	#N's	#mm	#short ind	#long ind	#mis.
	TW20 genome								
MICRA without ref	384	97610	99.111	2813 + 52 part	403	483	141	56	27
MICRA with ref	201	3043203	100.000	2890 + 0 part	0	2	0	0	13
SPAdes	55	144634	98.169	2826 + 27 part	0	98	9	7	1
	pTW20-1								
MICRA without ref	15	4608	84.523	12 + 8 part	19	13	0	0	0
MICRA with ref	6	18289	73.899	13 + 4 part	0	2	0	0	0
SPAdes	5	173232	71.702	13 + 3 part	0	0	0	0	0
	pTW20-2								
MICRA without ref	1	3138	100.000	0 + 2 part	0	0	0	0	0
MICRA with ref	2	3110	100.000	1 + 1 part	0	0	0	0	0
SPAdes	1	3138	100.000	0 + 2 part	0	0	0	0	0

Table S4: QUAST comparison of results obtained with MICRA and SPAdes

The sequences produced by MICRA are more broken up (greater number of contigs) than with *de novo* assembly with SPAdes due to the MICRA mapping strategy but the covered genome fraction is greater both for chromosome and plasmids and considering or not the reference sequences. Indeed, when the genome selected by MICRA shows an organization different from the studied genome, the homologous sequences are well identified by mapping strategy but their size are dependent of the size of these similar regions between studied and selected sequences, explaining the higher number of contigs produced by MICRA.

To compare the quality of annotations generated with MICRA and *de novo* based method, 3,174 CDSs were extracted from NCBI chromosome and plasmid entries. An in-house script was used to BLAST the reference CDSs against the CDSs predicted by MICRA (with and without reference) and by PROKKA from SPAdes contigs. Once a predicted CDS sequence was identified as present, it was removed from the list in order to count exactly the number of identified reference CDSs. A predicted CDS was considered as a true positive (TP) if it has a significant hit into the reference CDSs (reference and predicted CDSs have to be covered at least to 97%), as a false positive (FP) if it has not BLAST hit and as false negatives (FN) if the reference CDSs has no similar sequence predicted by the annotation tool. Results are shown in the top part of table S5 (part “no redundant CDSs”).

	# CDSs	True Positives	False Negatives	False Positives	precision	recall	F-measure
	no redundant CDSs						
MICRA without ref	3029	2798	282	231	0.92	0.91	0.92
MICRA with ref	3155	3068	12	87	0.97	1.00	0.98
SPAdes+PROKKA	2846	2776	304	70	0.98	0.90	0.94
	redundant CDSs						
MICRA without ref	3029	2975	282	54	0.98	0.91	0.95
MICRA with ref	3155	3145	12	10	1.00	1.00	1.00
SPAdes+PROKKA	2846	2782	304	64	0.98	0.90	0.94

Table S5: Precision, recall and F-measure values comparing MICRA and *de novo* approaches for sequence annotation.

All methods present high efficiency values (greater than 0.90 for precision, recall and F-measure). MICRA correctly identified more CDSs than the *de novo* based strategy (fewer FN) but showed more false-positive CDSs. The higher number of FPs was obtained when MICRA was run without the reference sequence (231 FPs). Checking these FP CDS, we noticed that they were not really false positives but redundant sequences. In order to take this discrepancy into account we decided to add an alternative definition of FP and TP CDs: a predicted CDS was considered as TP if it has a significant hit among the reference CDSs (without removing the corresponding reference CDS and so allowing redundant CDSs) and as FP when the predicted CDS has no significant BLAST hit against the reference CDS set. Results of this alternative definition are shown in the second part of table S5 (part “redundant CDSs”). Results from the *de novo* based approach did not significantly changed (304 FN and 64 FP) whereas they are highly different for MICRA. Only 10 FPs were identified when the reference sequence is used and 54 without reference sequence leading to a precision of 0.98 and a recall of 0.91 meaning that MICRA is able to correctly identify a large part of the CDSs. The relatively high number of redundant sequences is explained by the MICRA strategy. Figure S3 shows the schematic representation of sequence generation in MICRA with paired-end data.

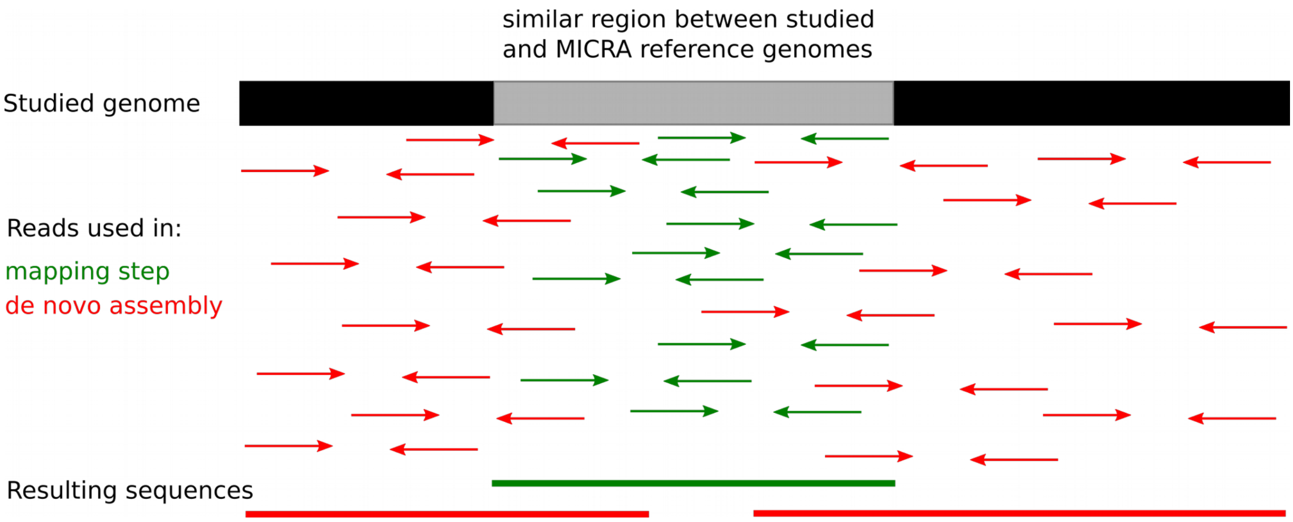

Figure S3: Schematic representation of sequence generation in MICRA with paired-end data

When the closest reference genome selected by MICRA present an organization different from the studied genomes, regions of high similarity are flanked by non-similar regions. During the mapping step, only concordant pairs (in green) are mapped against the selected genome producing a consensus sequence from mapping (in green). The read pairs which were not mapped concordantly are then used in the *de novo* assembly step leading to generation of additional sequences (in red). The read pairs localized at the junction between similar and non-similar regions (not-mapped) generate overlapping and redundant sequences with the ones generated from mapping.

To conclude this part, the results show that MICRA produced redundant sequences with paired-end data, especially with selected sequences genetically distant from the studied sequence, but also that MICRA is all the same able to correctly identify more true CDSs than other approaches.

Ion Torrent sequencing data

The strain *Staphylococcus aureus subsp. aureus* ST398 containing one chromosome (NCBI:AM990992.1) and three plasmids (pS0385-1 NCBI:AM990993.1, pS0385-2 NCBI:AM990994.1 and pS0385-3 NCBI:AM990995.1) was studied in [8]. Ion torrent data were available for this strain (SRA:ERR493467) containing 1,199,538 reads with a mean size of 250bp and a quality varying from Q18 to Q30. MICRA was run without providing any reference sequence. The closest genome identified by MICRA was *Staphylococcus aureus* subsp. Aureus ED98 (NCBI:NC_013450.1) covered at 92.2% with 73% of reads mapped and a mean depth of 22X. MICRA identified the pS0385-3 plasmid covered at 100% with a sequencing depth of 4840X and the *Staphylococcus aureus* plasmid pS194 (NCBI:X06627.1), similar to the pS0385-2 plasmid, covered at 100% with a mean depth of 4210X. The 86,965 reads still unmapped after the iterative step are then *de novo* assembled producing 120 contigs greater than 500bp. A complete *de novo* assembly was performed with MIRA and contigs were annotated with PROKKA. Table S6 shows the assembly comparison with QUAST.

	#contigs	N50	% genome	# genes	#N's	#mm	#short ind	#long ind	#mis.
	chromosome								
MICRA	288	40157	96.674	2511 + 87 part	389	6225	1278	128	35
MIRA + PROKKA	131	2373	7.175	151 + 96 part	6	54	162	1	1
	plasmid1								
MICRA	3	4021	97.217	3 + 1 part	0	2	2	0	0
MIRA + PROKKA	1	4753	90.583	3 + 0 part	0	0	1	0	0
	plasmid1								
MICRA	2	4398	100.000	3 + 2 part	0	10	15	1	0
MIRA + PROKKA	44	764	100.000	4 + 2 part	4	0	14	0	0
	plasmid3								
MICRA	2	3158	100.000	2 + 0 part	0	0	0	0	0
MIRA + PROKKA	41	696	100.000	1 + 1 part	13	0	14	0	0

Table S6: QUAST comparison of results obtained with MICRA and MIRA

This strain contains one chromosome and three plasmids which are present in variable copy number putting the MIRA assembler in an awkward position due to the difference of sequencing depth. MIRA *de novo* assembly required more than 24 hours (less than 30 minutes for MICRA) and obtained contigs covered only 7.1% of the chromosome sequence whereas MICRA managed to build more than 96.6% of the chromosome. The three plasmid sequences were more successfully built with both approaches.

	# CDSs	True Positives	False Negatives	False Positives	precision	recall	F-measure
MICRA	2850	2550	160	300	0.89	0.94	0.92
MIRA+PROKKA	711	223	2487	488	0.31	0.08	0.13

Table S7: Precision, recall and F-measure values comparing MICRA and *de novo* approaches for sequence annotation.

Analyzing the annotations (see Table S7) showed that MICRA is able to correctly identify CDSs with a precision of 0.89 and a recall of 0.94. On the other hand, considering the difficulty of the *de novo* assembly process, only 711 CDSs were predicted leading to a high number of FNs.

The results showed that MICRA is able to deal with a varying sequencing depth and can be of great interest in case of difficult assembly process.

4-2 Study of *Clostridium autoethanogenum* data

A draft genome for Clostridium autoethanogenum DSM10061 strain, an industrially relevant bacterium, was published by Bruno-Barcena et al. in 2013 [9] from 454 GS FLX and Ion Torrent PGM data. Pacific biosciences single-molecule DNA sequencing technology was used to generate a finished genome sequence by Brown *et al.* in 2014 [10]. Humphreys et al. in 2015 [11] inspected the Brown *et al.* closed genome sequence and identified some frame-shift mutations resulting in premature stop-codons. They re-sequenced this strain using Illumina MiSeq technology and observed 243 single nucleotide discrepancies when compared to the previous published genome due to sequencing errors and finally completed a comprehensive manual annotation. The study of this strain represented a very good application case for MICRA testing.

Ion Torrent sequencing data

The Ion torrent dataset (SRA:SRR1748018) contains 453,686 reads with a mean size of 270bp and a quality comprised between Q20 and Q34. MICRA was first run without reference sequence. The *Clostridium ljungdahlii* DSM 13528 genome (NCBI:NC_014328.1) was detected as closest reference genome covered at 87.8% with 86.8% of reads mapped and a mean depth of 16X. From

unmapped reads, 130 contigs greater than 500bp were generated at the *de novo* step.

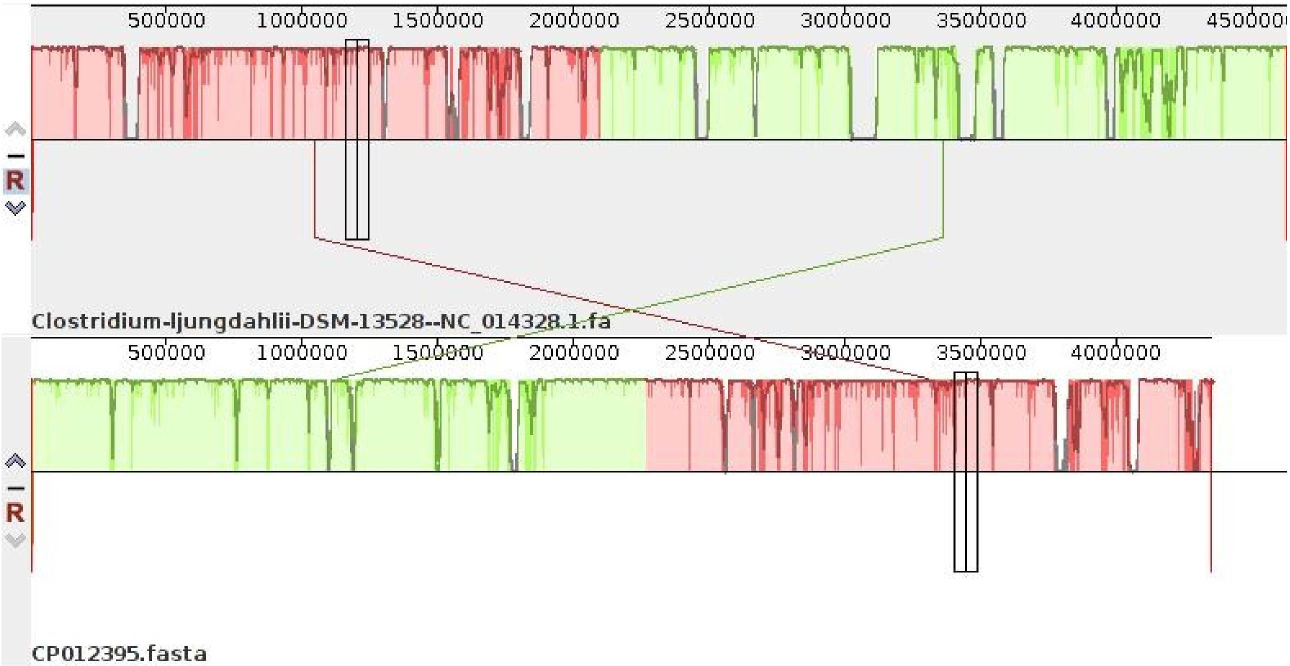

Figure S4: Comparison of *Clostridium ljungdahlii* DSM 13528 and *Clostridium autoethanogenum* DSM10061 genomes using MAUVE

Comparison of the DSM13528 and DSM10061 sequences (Figure S4) showed that these genomes share similar regions. The DSM13528 sequence is covered at 88% by DSM10061 sequence (identity: 99%) and respectively the DSM10061 is covered at 93% by DSM13528 sequence (identity: 99%) but with different sequence organization.

MICRA was also run with the *Brown et al.* finished genome as reference and a complete *de novo* assembly was performed with MIRA and contigs were annotated with PROKKA. Table S8 shows the assembly comparison obtained with QUAST considering the last finished sequence CP012395.1.

	#contigs	N50	% genome	# genes	#N's	#mm	#short ind	#long ind	#mis.
MICRA with ref	23	400580	99.944	3951 + 13 part	284	16	173	0	0
MICRA without ref	237	62097	99.565	3872 + 84 part	1077	3896	591	114	19
MIRA+PROKKA	146	77713	99.026	3900 + 62 part	42	130	1039	5	17

Table S8: QUAST comparison of results obtained with MICRA and MIRA

Both approaches identified more than 99% of the genome sequence. Sequences produced by MICRA without reference sequence are more broken up but cover more than 99.5% of the genome sequence.

	# CDSs	True Positives	False Negatives	False Positives	precision	recall	F-measure
MICRA with ref	4113	3932	32	181	0.96	0.99	0.97
MICRA without ref	3638	3552	442	86	0.98	0.89	0.93
MIRA+PROKKA	4611	3943	21	668	0.86	0.99	0.92

Table S9: Precision, recall and F-measure values comparing MICRA and *de novo* approaches for sequence annotation.

Table S9 shows the comparison of annotations produced by MICRA and MIRA approaches.

MICRA without reference sequence generated less CDSs than the other tools with the highest number of FNs but also the lowest number of FPs. The *de novo* based approach produced the highest number of FPs. MICRA used with Brown *et al.* genome sequence returns good values for precision and recall and return 87 variant calls among which 71 are true variations.

Illumina sequencing data

Illumina dataset (NCBI:SRR2969415) contains 1,798,962 read pairs of 250bp with a mean quality of Q38. MICRA was first without reference sequence. The *Clostridium ljungdahlii* DSM13528 sequence (NCBI:NC_014328.1), the same than the one identified with Ion torrent data, was selected as the closest reference sequence, covered at 87% with 86% of reads mapped and a mean depth of 167X. 1,855 contigs greater than 500 bp were produced in the *de novo* step. MICRA was also run with the Brown *et al.* genome sequence as reference and finally reads were assembled with SPAdes and the resulting contigs were annotated with PROKKA. Results of assemblies are compared with QUAST against the last genome sequence (CP012395.1) and are given in Table 10.

	#contigs	N50	% genome	# genes	#N's	#mm	#short ind	#long ind	#mis.
MICRA with ref.	2260	4352448	100.000	3964 + 0 part	0	5	1	8	152
MICRA without ref.	1940	40896	99.894	3944 + 16 part	128	743	153	65	131
SPAdes+PROKKA	37	397010	98.908	3943 + 13 part	0	95	2	2	0

Table S10: QUAST comparison of results obtained with MICRA and SPAdes

MICRA allows to cover more than 99.8% of the genome sequence and the *de novo* based approach more than 98.9%. We then studied the quality of annotations generated by the two approaches (Table S11).

	# CDSs	True Positives	False Negatives	False Positives	precision	recall	F-measure
	no redundant CDSs						
MICRA with ref.	4582	3938	26	644	0.86	0.99	0.92
MICRA without ref.	4323	3595	369	728	0.83	0.91	0.87
SPAdes+PROKKA	4014	3943	21	71	0.98	0.99	0.99
	redundant CDSs						
MICRA with ref.	4582	4525	26	57	0.99	0.99	0.99
MICRA without ref.	4323	4262	369	71	0.98	0.92	0.95
SPAdes+PROKKA	4014	3947	21	67	0.98	0.99	0.99

Table S11: Precision, recall and F-measure values comparing MICRA and *de novo* approaches for sequence annotation.

Without reference sequence, MICRA showed the highest number of FNs (369 whereas the number is less than 30 with the *de novo* or MICRA with Brown *et al.* genome sequence). However, taking into account the redundant CDSs, the results show that MICRA, even without the reference sequence, is able to identify a higher number of TP CDSs than the *de novo* based approach.

A very interesting result returned by MICRA when used with Brown *et al.* genome sequence is the list of variant calls. Indeed, MICRA returned exactly the 242 published differences (only the A insertion at position 1 was not identified).
These results greatly illustrate the interest and the power of the use of MICRA in re-sequencing projects or mutants study projects.

5- Parameters used in this study

All the results presented in this manuscript are available at http://www.pegase-biosciences.com/MICRA/data.html. The MICRA parameters used for each dataset are summarized in table S12**.**

Experiment	reads	Technology	Pre-process	Ref. seq.	%plasmid	low cov.	min freq.	repeats	%CDS	antibio
Default parameters			no	automatic	70 %	5	90 %	no	80 %	no
DH10B										
simulated IT	simulated	Ion Torrent	no	automatic	60 %	5	90 %	no	80 %	yes
simulated PE	simulated	Illumina	no	automatic	60 %	5	90 %	no	80 %	yes
real subset	real run*	Ion Torrent	no	ID list	60 %	5	90 %	no	80 %	yes
real data	real run	Ion Torrent	no	ID list	60 %	5	90 %	no	80 %	yes
real data without ref.	real run	Ion Torrent	no	automatic	60 %	5	90 %	no	80 %	yes
P134 strain of Bordetella pertussis										
P134 strain	SRR4019415	Ion Torrent	cutadapt	ID list	60 %	3	90 %	yes	80 %	yes
Escherichia coli O104:H4										
TY2482 with ref.	5 runs	Ion Torrent	no	ID list	60 %	3	90 %	no	80 %	yes
TY2482 without ref.	5 runs	Ion Torrent	no	automatic	60 %	3	90 %	no	80 %	yes
2009-2050 strain	SRR647664*	Illumina	no	ID list	60 %	5	90 %	no	80 %	yes
2009-2071 strain	SRR647666*	Illumina	no	ID list	60 %	5	90 %	no	80 %	yes
Staphylococcus aureus										
PE data	ERR1274626	Illumina	cutadapt	ID list	60 %	5	90 %	no	90 %	yes
PE data without ref.	ERR1274626	Illumina	cutadapt	automatic	60 %	5	90 %	no	90 %	yes
IT data	ERR493467	Ion Torrent	cutadapt	automatic	60 %	5	90 %	no	80 %	yes
Clostridium autoethanogenum										
PE data	SRR2969415	Illumina	cutadapt	ID list	70 %	5	80 %	no	90 %	no
PE data without ref.	SRR2969415	Illumina	cutadapt	automatic	70 %	5	80 %	no	90 %	no
IT data	SRR1748018	Ion Torrent	cutadapt	ID list	70 %	5	80 %	no	90 %	no
IT data without ref.	SRR1748018	Ion Torrent	cutadapt	automatic	70 %	5	80 %	no	90 %	no

Table S12: MICRA parameters used in this study.
The first line shows the values of MICRA default parameters. The first column describes the experiment. The “reads” column gives the dataset which can be downloaded from SRA or the MICRA website. Datasets marked with an asterisk mean that a subset of the reads was used. The “Technology” column gives the sequencing technology parameter. The “Pre-process” column indicates when the pre-processing module was run with cutadapt for quality and adapter trimming (default parameters). The “Ref. seq” column indicates if the selection of reference sequences was done automatically or fixed with an ID list. All the ID lists used here are available on the MICRA website. The “%plasmid” column indicates the percentage of covered sequence for a plasmid to be considered in the analysis. The “low. cov.” is the low coverage threshold parameter which corresponds to the minimum number of reads mapped at a given position in the reference sequence for which the position is considered as covered. The “min. freq” column corresponds to the minimum frequency from which a variant is called. The “repeat” column indicates when the option “high repeat content” was selected meaning that the mapper parameters will be tuned to deal with repeats. The “%CDS” column gives the minimum CDS coverage in BLAST step **(**during the contig annotation, a BLAST against the PATRIC CDS databank is performed). Finally, the last column indicates when the antibiotic module was performed (with default parameter setting).

Note that for all experiments the SAM files were kept by checking the corresponding box on MICRA interface.

6- Additional references

1. Martin M. Cutadapt removes adapter sequences from high-throughput sequencing reads. EMBnet.journal. 2011;17:10.

2. Herrou J, Bompard C, Wintjens R, Dupré E, Willery E, Villeret V, et al. Periplasmic domain of the sensor-kinase BvgS reveals a new paradigm for the Venus flytrap mechanism. Proc. Natl. Acad. Sci. U. S. A. 2010;

3. Uhl MA, Miller JF. Central role of the BvgS receiver as a phosphorylated intermediate in a complex two-component phosphorelay. J. Biol. Chem. 1996;

4. Rohde H, Qin J, Cui Y, Li D, Loman NJ, Hentschke M, et al. Open-source genomic analysis of Shiga-toxin-producing E. coli O104:H4. N. Engl. J. Med. 2011;365:718–24.

5. Andrews S. FastQC. Available from: http://www.bioinformatics.babraham.ac.uk/projects/fastqc/

6. Ahmed SA, Awosika J, Baldwin C, Bishop-Lilly KA, Biswas B, Broomall S, et al. Genomic Comparison of Escherichia coli O104:H4 Isolates from 2009 and 2011 Reveals Plasmid, and Prophage Heterogeneity, Including Shiga Toxin Encoding Phage stx2. PLoS One. 2012;7.

7. Holden MTG, Lindsay JA, Corton C, Quail MA, Cockfield JD, Pathak S, et al. Genome sequence of a recently emerged, highly transmissible, multi-antibiotic- and antiseptic-resistant variant of methicillin-resistant Staphylococcus aureus, sequence type 239 (TW). J. Bacteriol. 2010;

8. Kaas RS, Leekitcharoenphon P, Aarestrup FM, Lund O. Solving the problem of comparing whole bacterial genomes across different sequencing platforms. PLoS One. 2014;

9. Bruno-Barcena JM, Chinn MS, Grunden AM. Genome Sequence of the Autotrophic Acetogen Clostridium autoethanogenum JA1-1 Strain DSM 10061, a Producer of Ethanol from Carbon Monoxide. Genome Announc. 2013;

10. Brown SD, Nagaraju S, Utturkar S, De Tissera S, Segovia S, Mitchell W, et al. Comparison of single-molecule sequencing and hybrid approaches for finishing the genome of Clostridium autoethanogenum and analysis of CRISPR systems in industrial relevant Clostridia. Biotechnol. Biofuels. 2014;

11. Humphreys CM, McLean S, Schatschneider S, Millat T, Henstra AM, Annan FJ, et al. Whole genome sequence and manual annotation of Clostridium autoethanogenum, an industrially relevant bacterium. BMC Genomics. 2015;
